# The Non-N^6^-Methyladenosine Epitranscriptome Patterns and Characteristics of Tumor Microenvironment Infiltration and Mesenchymal Transition in Glioblastoma

**DOI:** 10.3389/fimmu.2021.809808

**Published:** 2022-01-26

**Authors:** Jianye Xu, Zijie Gao, Kaining Liu, Yang Fan, Zongpu Zhang, Hao Xue, Xing Guo, Ping Zhang, Lin Deng, Shaobo Wang, Huizhi Wang, Qingtong Wang, Rongrong Zhao, Gang Li

**Affiliations:** ^1^ Department of Neurosurgery, Qilu Hospital, Cheeloo College of Medicine and Institute of Brain and Brain-Inspired Science, Shandong University, Jinan, China; ^2^ Shandong Key Laboratory of Brain Function Remodeling, Jinan, China

**Keywords:** glioblastoma, non-m^6^A RNA modification, tumor microenvironment, mesenchymal transition, immunotherapy

## Abstract

**Background:**

An increasing number of RNA modification types other than N^6^-methyladenosine (m^6^A) modification have been detected. Nonetheless, the probable functions of RNA modifications beyond m^6^A in the tumor microenvironment (TME), mesenchymal (MES) transition, immunotherapy, and drug sensitivity remain unclear.

**Methods:**

We analyzed the characteristics of 32 non-m^6^A RNA modification regulators in 539 glioblastoma (GBM) patients and the TME cell infiltration and MES transition patterns. Using principal component analysis, a non-m^6^A epitranscriptome regulator score (RM score) model was established. We estimated the association between RM score and clinical characteristics, TME status, GBM subtypes, and drug and immunotherapy response.

**Results:**

Three definite non-m^6^A RNA modification patterns associated with diverse biological pathways and clinical characteristics were identified. The high RM score group was characterized by a poor prognosis, enhanced immune infiltration, and MES subtype. Further analysis indicated that the high RM score group had a lower tumor mutation burden as well as a weaker response to immunotherapy. The higher RM score group may benefit more from drugs targeting the EGFR and WNT signaling pathways.

**Conclusion:**

Our study exposed the potential relationship of non-m^6^A RNA modification regulators with clinical features, TME status, and GBM subtype and clarified its therapeutic value.

## Introduction

Glioblastoma (GBM) is the most ordinary malignant intracranial cancer (1). Patients with GBM have an approximately 14–16-month median survival time, and GBM is highly resistant to standard therapies because of its extraordinary immunosuppressive microenvironment and mesenchymal (MES) transition (2–4).

There are over 160 posttranscriptional RNA modifications (5). RNA modifications modulate the structure as well as functions of RNAs, which results in several diseases, including GBM (6, 7). In eukaryotic cells, the most common RNA modification is N^6^-methyladenosine (m^6^A) (8, 9). Intensive investigations have indicated that m^6^A facilitates a broad range of critical functions, including embryogenesis, neurogenesis, hematopoiesis, and tumorigenesis (10–13). Indeed, an increasing number of RNA modifications beyond m^6^A have been found and studied with the development of technology (14). We found that some RNA modifications have been recently reported to be widespread on mammalian RNA; these include pseudouridine (Ψ), N^6^,2′-O-dimethyladenosine (m^6^A_m_), N^1^-methyladenosine (m^1^A), alternative polyadenylation (APA), N^7^-methylguanosine (m^7^G), 2′-O-methylated nucleotides (N_m_), 5-methylcytidine (m^5^C), N^4^-acetylcytidine (ac^4^C), adenosine-to-inosine RNA editing (I), and cytidine-to-uridine RNA editing (U) (14–16).

Ψ, the most abundant non-m^6^A modification, is generated by uridine isomerization (17). Ψ “writers” include TRUB1, TRUB2, PUS1, PUS7, and DKC1 (18). Ψ is required for proper folding and translation of transfer RNA (tRNA), ribosomal RNA (rRNA) structure stabilization, small nuclear ribonucleoprotein (snRNP) biogenesis, and messenger RNA (mRNA) splicing (19–21).

Detected at an approximately 10-fold lower m^6^A level, m^1^A is involved in the methylation of the adenine base on the first nitrogen atom as well as carries positive electricity (22). The m^1^A methylation process is mediated by methyltransferases (writers) consisting of TRMT61B, TRMT6, and TRMT61A, while the removal process is catalyzed *via* demethylases (erasers) containing ALKBH3, ALKBH1, and FTO (23–25). Various specific RNA-binding proteins (readers), including YTHDC1, YTHDF3, YTHDF2, and YTHDF1, can recognize the m^1^A motif, thus affecting m^1^A functions (26). m^1^A is necessary for tRNA structure stabilization, proper rRNA biogenesis, and methylated mRNA translation (22, 27). If 2′-O-methyladenosine is the first ribotide behind the m^7^G cap of mRNA, it could be methylated at the N^6^ position to render m^6^A_m_, which is related to mRNA metabolism (28, 29). PCIF1 is the writer that creates the m^6^A_m_ modification, and FTO is the eraser of m^6^A_m_ (25, 30). APA cleaves mRNA at more than one site and adds poly (A) tails to generate different transcripts of the 3′-untranslated region (3′UTR) or coding regions, which regulates the function, stability, and translation efficiency of RNAs (31). PABPN1, NUDT21, CLP1, PCF11, and CPSF6 can regulate the APA process (32).

At levels similar to those of m^1^A, m^7^G, a modification carrying a positive charge at the 5′ cap, is necessary for mRNA export, splicing, and translation (33). In addition, the METTL1-catalyzed m^7^G tRNA methylome is important for the translation of mRNA (34). N_m_ is relatively widespread in rRNA, tRNA, snRNA, and microRNA and can be installed by FBL (35, 36). In addition, N_m_ is essential for adjusting the structure and function of ribosomes (37).

Appearing in both coding and non-coding regions, m^5^C sites are chiefly CG-rich regions that accumulate in the UTRs of mRNA (38). The writer of m^5^C is NSUN2, while the eraser is TET2 (14, 39, 40). In addition, YBX1 has been reported to be an m^5^C reader (41). m^5^C modification plays an essential role in mRNA export, tRNA structure stabilization, and rRNA translational fidelity (39, 42). Catalyzed by NAT10 and THUMPD1, ac^4^C was subsequently discerned in serine and leucine 18S rRNA and tRNAs. ac^4^C, the sole acetylation modification in eukaryotic RNA, regulates mRNA translation and ribosome biogenesis (43, 44).

RNA editing is a kind of programmed posttranscriptional mechanism altering nucleotides in selected transcripts (45). Catalyzed by ADAR, ADARB1, and ADARB2, RNA editing can transform the sequence as well as alter transcriptional procedures (46). Accumulating in 3′UTRs, U RNA editing alters the protein level (47). UPP1 can catalyze the phosphorolysis of uridine to uracil (48, 49).

To thoroughly comprehend the significance of RNA modifications beyond m^6^A, investigation of the crosstalk among diverse patterns of RNA alterations is urgently needed. Ten kinds of non-m^6^A RNA modification “regulators” may build a complex and significant regulatory network in GBM, which might help elucidate GBM tumorigenesis mechanisms.

Immune checkpoint blockade therapy (ICB therapy), also known as immunotherapy, has delivered promising clinical outcomes for various cancers; nevertheless, it commonly exhibits a poor response due to the tumor microenvironment (TME) (50, 51). Unfortunately, a large number of patients with GBM have not experienced outstanding survival benefits; that is, immunotherapy for GBM is far from reaching clinical expectations (52). Accordingly, to enhance the effect of immunotherapy for GBM, it is crucial to thoroughly investigate the immunosuppressive TME. Recent studies have revealed that m^6^A plays a significant role in complex TME formation (53, 54). Estimating m^6^A patterns of a type of tumor to characterize TME infiltration could guide more effective immunotherapy strategies (55, 56). Non-m^6^A RNA modification as well as correlative regulators are highly related to the microenvironment of immune cells as well as tumor cells. TET2 deficiency in Treg cells results in T-cell activation (57). NAT10 regulates the function of CD4^+^ T cells (58). However, few studies have systematically analyzed the non-m^6^A RNA modification patterns of individual tumors, while these RNA modifications play a non-negligible role in tumorigenesis (16, 59).

Based on genetic transcription signatures, GBM can be categorized into three subtypes (MES; classical, CL; as well as proneural, PN) (3). MES transition promotes radiochemotherapy resistance in GBM (60, 61). The MES subtype is particularly aggressive among these three subtypes, while the PN subtype yields the best prognosis (62, 63). In addition, the MES-subtype GBM promotes the formation of an immunosuppressive TME, while the TME advances the MES transition in GBM (62, 64). An increasing number of studies have demonstrated that non-m^6^A RNA modification regulators are significantly correlated with MES transition. It has been reported that ADAR and YBX1 promote MES transition by regulating oncogenic microRNA maturation (65, 66). Nevertheless, research on RNA modifications beyond m^6^A is still not as mature as that for m^6^A for many reasons, including technology limitations. Accordingly, it is significant to investigate the non-m^6^A epitranscriptome patterns and characteristics of TME infiltration and MES transition in GBM.

In this study, we explored somatic mutation data for 390 GBM cases from The Cancer Genome Atlas (TCGA, http://portal.gdc.cancer.gov/; http://xena.ucsc.edu/) and integrated gene expression data from 539 GBM samples from TCGA and Chinese Glioma Genome Atlas (CGGA, https://www.cgga.org.cn/) cohorts to evaluate the RNA modification patterns. We discovered that non-m^6^A RNA modification patterns were associated with TME cell-infiltrating characteristics as well as MES transition. Next, according to differentially expressed genes (DEGs), we established a non-m^6^A epitranscriptome “regulator” score (RM score) to evaluate the effect on the non-m^6^A epitranscriptome in individual patients. Eventually, we verified the pertinence of the RM score in distinguishing the posttranscriptional and transcriptional events as well as evaluated its importance in predicting the response to targeted and ICB therapy.

## Materials and Methods

### Data Collection and Processing

The study workflow chart is shown in **Figure S1A**. Genetic transcription data as well as clinical information on GBM patients were obtained from TCGA and the CGGA. Somatic mutation counts and copy number variation (CNV) were obtained from the TCGA database. In total, 539 GBM samples (CGGA_batch_1:139, CGGA_batch_2:249, TCGA:151) were gathered in this study for further analyses. We used sva package’s “ComBat” algorithm to correct non-biotechnology deviations causing batch effects. R Bioconductor packages and R (version 4.10) were used to analyze the data.

### Unsupervised Clustering for 32 Non-m^6^A RNA Modification Regulators

We extracted 32 non-m^6^A RNA modification regulators and their expression from the TCGA and CGGA databases. These non-m^6^A RNA modification regulators included 10 RNA modification types, which are listed in **Table S1**. Using unsupervised cluster analysis, different non-m^6^A RNA modification patterns concerning 32 non-m^6^A RNA modification regulatory factors were identified, and the patients were classified into different clusters. We applied the Consensus Cluster Plus package to execute the steps above with1,000 repetitions to guarantee classification stability.

### Gene Functional Annotation Based on Gene Set Variation Analysis

To analyze the differences in biological processes between non-m^6^A RNA modification patterns, gene set variation analysis (GSVA) enrichment was performed *via* the “GSVA” R package. We downloaded the gene set “c5.go.bp.v7.4” as well as “c2.cp.kegg.v7.4” from the Molecular Signatures Database (MSigDB, https://www.gsea-msigdb.org/gsea/msigdb/index.jsp) for GSVA.

### TME Cell Infiltration Estimation

First, single-sample gene set enrichment analysis (ssGSEA) was used to identify tumor immune-infiltrating cell abundance in GBM samples. The markers of 28 immune-related cells and types were obtained from the dataset of Bindea et al. (67). Using an unsupervised hierarchical clustering algorithm, 539 GBM samples were assigned to two groups based on immune infiltration. Next, ESTIMATE was used to calculate the immune score as well as stromal score (68). Then, we used CIBERSORT (http://cibersort.stanford.edu/) to measure the infiltration levels of 22 distinct immune cells among the GBM samples.

### Estimation of the MES/PN Score

Verhaak et al. identified three prognostic subtypes by integrated genomic analysis (69). There are significant prognostic differences between different subtypes, and accurate identification of subtypes can also guide treatment strategies. We downloaded the gene sets “VERHAAK_GLIOBLASTOMA_MESENCHYMAL” and “VERHAAK_GLIOBLASTOMA_PRONEURAL” from the MSigDB database v7.4 and quantified the MES and PN scores of the GBM samples using the ssGSEA algorithm.

### Identification of DEGs Among Non-m^6^A RNA Modification Patterns

To identify non-m^6^A RNA modification-related genes, DEGs among three different non-m^6^A RNA modification characteristics were arranged *via* the limma R package. The significance criterion for DEGs was *p*-value <0.01. To evaluate the function of DEGs, Gene Ontology (GO) analysis was performed to calculate biological process (BP), molecular function (MF), and cellular component (CC) terms using DAVID (https://david.ncifcrf.gov/) with a *p*-value cutoff of <0.05.

### Cell Lines and Cell Culture

U-118 MG (Chinese Academy of Sciences Cell Bank) and LN-229 (ATCC Cell Bank) cell lines were cultured in DMEM (Sigma, USA) supplemented with 10% FBS (Thermo Fisher Scientific, USA). THP-1 cells (Chinese Academy of Sciences Cell Bank) were cultured in RPMI-1640 (Sigma) supplemented with 10% FBS. The cell lines were incubated at 37°C with 5% CO_2_. To induce their differentiation into macrophages, THP-1 cell lines were incubated with 100 ng/ml phorbol 12-myristate 13-acetate (PMA, Sigma) for 24 h.

### Small Interfering RNA and Plasmid Transfection

Small interfering RNAs (siRNAs) targeting UPP1 and plasmid overexpression of FTO were synthesized (GenePharma, China). SiRNAs and plasmids were transfected with Lipofectamine™ 3000 reagent (Thermo Fisher Scientific) according to the protocol of the manufacturer.

### Western Blotting

Protein was extracted from GBM cell lines. The blots were incubated with primary antibodies against UPP1 (Abcam, UK), FTO (Cell Signaling Technology, USA), and ACTIN (Cell Signaling Technology).

### 5-Ethynyl-2′-deoxyuridine Cell Proliferation Assay

A 5-ethynyl-2′-deoxyuridine (EdU) cell proliferation assay kit (RiboBio, #C10310-1; China) was used to evaluate the proliferative activity of GBM cells. The cell proliferation rate was assessed *via* the ratio of EdU-positive (red) cells to total Hoechst-positive (blue) cells.

### Transwell Assay

To evaluate migratory ability, GBM cells were added to the top chamber in DMEM without FBS, and the bottom chamber was filled with 10% FBS DMEM. To measure the ability to recruit macrophages, macrophages were added to the top chamber in RPMI-1640 without FBS, and the bottom chamber was filled with 10% FBS RPMI-1640 and growth media of different groups of GBM cells. After 24 h of incubation, the membrane was fixed in 4% paraformaldehyde and stained with crystal violet.

### Constructing the RM Scoring System to Evaluate Individual GBM Samples

First, overlapping DEGs distinguished from non-m^6^A RNA modification clusters were chosen for analysis. Then, we analyzed the prognostic value for each overlapping DEG *via* a univariate Cox regression model, and significant prognostic genes were identified. Next, the PCA algorithms were used to generate the RM score. Both principal component 2 and principal component 1 were selected to describe the RM scoring system.


$$\text{RM score}=\sum {PC2}_{i}-{PC1}_{i}$$

where *i* is the DEG expression.

### Immunofluorescence

Tumor tissues were obtained from 12 patients treated for GBM at Qilu Hospital. RNA quantity and quality were estimated *via* an Agilent 2100 bioanalyzer (Agilent Technologies) and a Nanodrop 2000 spectrophotometer (Thermo Fisher Scientific). The mRNA library was prepared by using the NEBNext^®^ Ultra™ RNA Library Prep Kit (Beijing Novel Bioinformatics Co., Ltd.). The HiSeqX platform (Illumina) on the Illumina standard protocol was used for RNA sequencing. Next, we performed a PCA algorithm based on the expression of DEGs to calculate the RM scores of the 12 GBM patients. We selected three cases with the highest as well as three cases with the lowest scores and performed immunofluorescence (IF) staining. After blocking with 5% goat serum (Gibco, USA) for 30 min, samples were incubated with primary antibodies at 4°C overnight. The following primary antibodies were used: rabbit SOX2 antibody (3579, Cell Signaling Technology, 1:400, USA), mouse CD44 antibody (3570, Cell Signaling Technology, 1:400), mouse CD163 antibody (ab156769, Abcam, 1:100, UK), and rabbit FoxP3 antibody (12653, Cell Signaling Technology, 1:400). Next, the samples were incubated with fluorophore-conjugated secondary antibodies at 37°C for 1 h. Alexa Fluor 594 anti-rabbit antibody (A11037; Invitrogen; 1:400) and Alexa Fluor 488 anti-mouse antibody (A11029; Invitrogen; 1:400) were used. DAPI was used to stain the cell nuclei. All immunostained samples were analyzed using Leica Application Suite Software and a Leica TCS SP8 confocal system. ImageJ was used to analyze the fluorescence intensity.

### Summary of Genomic and Clinical Information of the Immunotherapy Cohort

We analyzed gene expression-related clinical information of the immunotherapy cohort and identified two independent immunotherapy cohorts: advanced urothelial tumor with atezolizumab treatment (IMvigor210 cohort, http://research-pub.gene.com/IMvigor210CoreBiologies/packageVersions/) and metastatic melanoma with pembrolizumab treatment (GSE78220 cohort). We obtained the expression data and clinical annotations of the GSE78200 cohort from the Gene Expression Omnibus (GEO) database.

### Association Analysis Between Drug Sensitivity and RM Score

Cancer Cell Line Encyclopedia (CCLE) RNA-seq data were obtained from https://portals.broadinstitute.org/ccle/. Cancer cell line drug responses, measured as area under the curves (AUCs) and drug pathways, were obtained from Genomics of Drug Sensitivity in Cancer (GDSC; https://www.cancerrxgene.org/downloads). Spearman correlation analysis was applied to estimate the association between drug sensitivity and RM score.

### Comparison of the m^6^A Score and the RM Score

The Bayesian information criterion (BIC) as well as Akaike information criterion (AIC) was applied to compare the two models. A model with lower BIC and AIC values was identified as a better model.

### RM Score Among Clinical Traits in Pan-Cancer

Univariate Cox regression analysis was used to investigate the time-dependent prognostic value of RM score in Pan-cancer. Overall survival (OS) and progression-free survival (PFS) were selected to study the relationship between RM score and prognosis. In addition, the correlation between RM score and 22 immune cell infiltration was calculated in 33 cancers, respectively.

### Statistical Analysis

The statistical analyses were generated by R-4.0.1. Distance and Spearman correlation analyses were applied to calculate 32 RNA modification expression correlation coefficients. Kruskal–Wallis and one-way ANOVA tests were applied to appraise difference comparisons of more than three collections. To assess the relationship between patient survival and the RM score, the optimal cutoff point for survival information for each dataset was determined using the survminer package. The log-rank test and Kaplan–Meier analysis were applied to assess survival among different clusters as well as RM score groups. The hazard ratio (HR) of DEGs was calculated *via* a univariate Cox regression model. To evaluate whether the RM score serves as an independent predictor for survival, sex, age, and isocitrate dehydrogenase (IDH) status were considered variables in the multivariate Cox regression model analysis. Statistical analysis was two-sided, as well as *p <*0.05 was regarded as statistically significant.

## Results

### Landscape of Genetic Variations in Non-m^6^A RNA Modification Regulators in GBM

**Figure 1:**
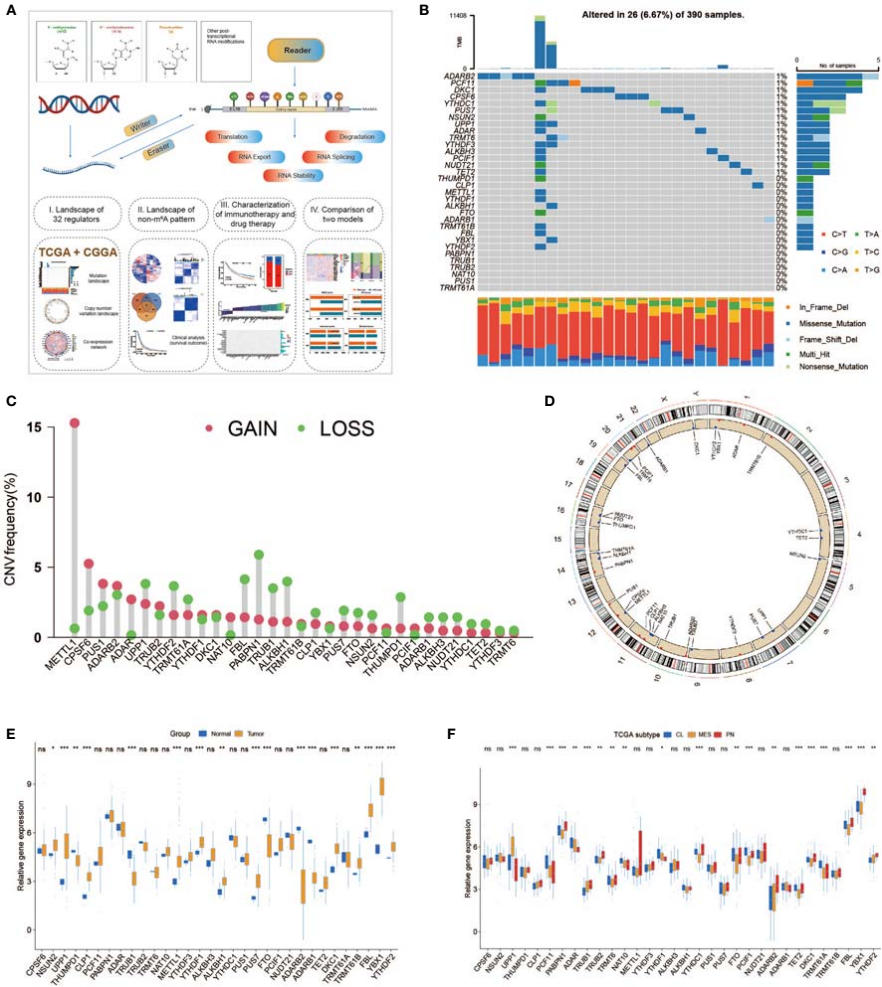
Expression and mutational data landscape of 32 non-m^6^A RNA-modified regulators in glioblastoma (GBM). **(A)** Landscape of this study workflow. **(B)** Mutation frequency of 32 non-m^6^A RNA modification genes in 390 GBMs from The Cancer Genome Atlas (TCGA) database. **(C)** CNV variation frequency of non-m^6^A RNA modification genes in TCGA-GBM. **(D)** The location of non-m^6^A RNA modification genes on 23 chromosomes in TCGA-GBM. **(E)** Expression of 32 non-m^6^A RNA modification genes between normal and GBM patients. **(F)** Expression of 32 non-m^6^A RNA modification genes in different TCGA GBM subtypes. * means P<0.05; ** means P<0.01; *** means P<0.001; ns means P>0.05.

A total of 32 non-m^6^A RNA modification regulators (**Table S1**) were included in the current study. **Figure 1A** shows that these non-m^6^A RNA modification regulators can dynamically and reversibly add, remove, and recognize non-m^6^A RNA-modified sites and potentially change biological functions, such as splicing, stability, export, translation, and degradation of RNA. We first summarized the somatic mutation prevalence of 32 non-m^6^A RNA modification regulators among GBM. Among 390 samples, 24 showed genetic alterations of non-m^6^A RNA modification regulators, with a frequency of 6.67% (**Figure 1B**). These mutations were multifarious and included splicing-related mutations, missense mutations, and deletions (**Figure 1B**). The mutation frequency of ADARB2 was the highest, while PABPN1, TRUB1, TRUB2, NAT10, PUS1, and TRMT61A showed no mutations among the 390 GBM patients (**Figure 1B**). Further analyses revealed mutation co-occurrence relationships among YTHDF2, YTHDF1, TRMT61B, THUMPD1, METTL1, FTO, FBL, YTHDF3, and UPP1 (**Figure S1B**). Moreover, the investigation of 32 non-m^6^A RNA modification regulators exhibited that CNV-related mutations were widespread. METTL1, CPSF6, PUS1, ADARB2, ADAR, and UPP1 showed widespread CNV amplification, while PABPN1, FBL, ALKBH1, and TRUB1 had CNV deletions (**Figure 1C**). The CNV alteration locations of 32 non-m^6^A RNA modification regulators on chromosomes are presented in **Figure 1D**. To determine whether genetic variations affected non-m^6^A RNA modification regulator expression among GBM cases, we analyzed the expression of these regulators between GBM and normal samples to determine whether CNV alterations could result in perturbations in non-m^6^A RNA modification regulator expression. Non-m^6^A RNA modification regulators with CNV amplification demonstrated substantially higher expression in GBM tissues than in normal brain tissues, while those with deletions exhibited the opposite trend (**Figures 1C, E**). Moreover, we determined that the regulator expression patterns significantly varied among the three subgroups (**Figure 1F**). O^6^-methylguanine DNA methyltransferase (MGMT) methylation, isocitrate dehydrogenase 1 (IDH1) mutation, and chromosomal 1p/19q codeletion have been found to confer a favorable prognosis in GBM (70). The same phenotypes were investigated in groups classified based on molecular subtypes (**Figures S1D–F**). Based on the expression of 32 non-m^6^A RNA modification regulators, we distinguished patients with GBM from normal controls (**Figure S1G**). The analyses revealed high genetic and transcriptomic landscape heterogeneity among non-m^6^A regulators between GBM and normal cases, indicating that genetic variations and expression alterations among non-m^6^A RNA modification genes play an essential role in GBM progression and occurrence.

### Non-m^6^A RNA Modification Patterns Mediated by 32 Regulators

To obtain an overall understanding of the expression pattern, 539 GBM cases from the TCGA and CGGA datasets that contained clinical information and OS data were enrolled. A univariate Cox regression model revealed that 10 of 32 non-m^6^A regulators were associated with the survival of GBM patients (**Figure S1C**).

**Figure 2:**
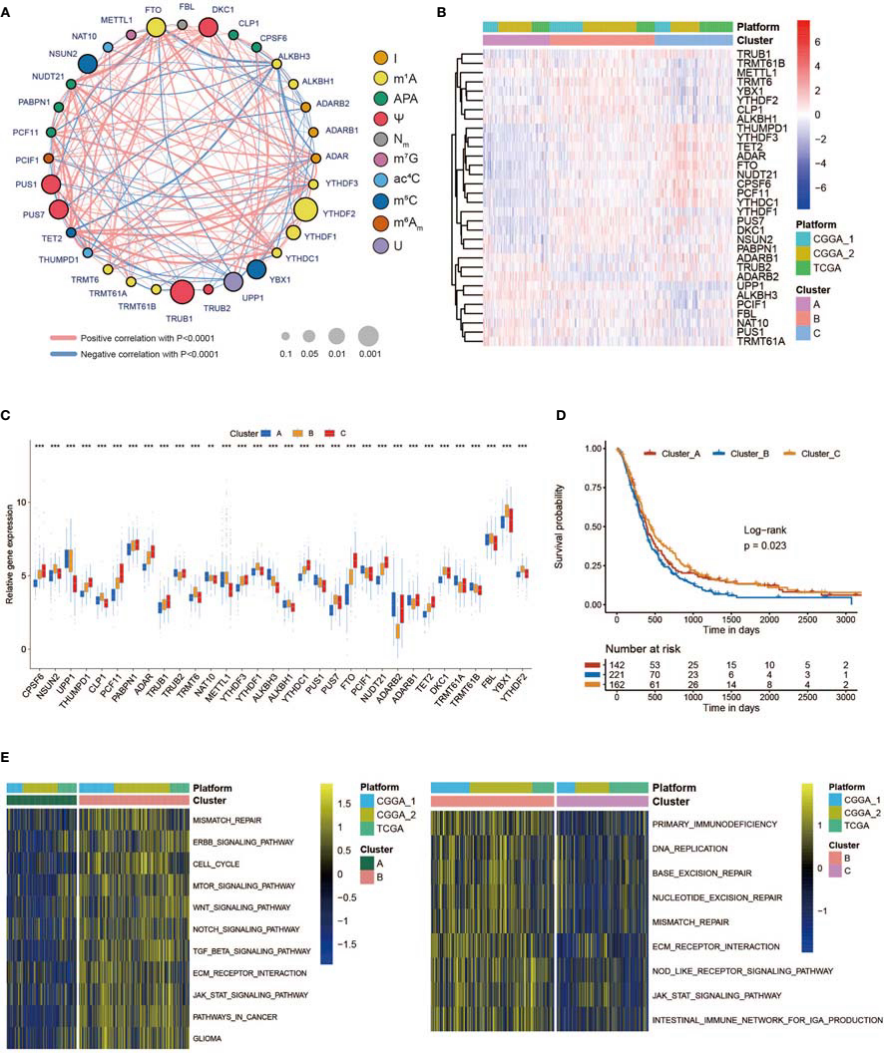
Three RNA modification patterns of non-m^6^A genes and relative biological functions. **(A)** Network plot showing the interaction between non-m^6^A RNA modification genes in GBM. The size of the circle represents the *p*-value of each gene for the survival prognosis. Black dots represent hazard survival factors, and green dots represent favorable survival factors. The thickness of the lines represents the correlation value between genes. The red and blue lines represent positive and negative correlations, respectively. Ten RNA modification types are marked with different colors. **(B)** Heatmap showing the unsupervised clustering of 32 non-m^6^A RNA modification regulators in 539 GBM patients. Each column represents a patient, and each row represents a non-m^6^A RNA modification regulator. **(C)** Expression of 32 non-m^6^A RNA modification genes between three non-m^6^A RNA modification patterns. **(D)** Kaplan–Meier curves showing the survival information of three non-m^6^A RNA modification patterns. **(E)** Heatmap showing the results of GSVA enrichment between different non-m^6^A RNA modification patterns. * means P<0.05; *** means P<0.001.

To investigate the relationships among regulators, we calculated pairwise correlations among the expression of the 32 regulators in the GBM cases (**Figure S2A**). We identified that the expression of DKC1, YBX1, TRMT6, and PUS7 was positively correlated with that of many other regulators, while the expression of UPP1 and ALKBH3 was negatively correlated with that of other regulators (**Figure S2A**). In addition, the protein–protein interactions between non-m^6^A RNA modification regulators are shown in **Figure S1H**. The comprehensive landscape of the intricate associations between non-m^6^A RNA modification regulators and the prognostic value for GBMs was visualized with the non-m^6^A RNA modification regulator network (**Figure 2A**; **Figure S2A**). We identified significant correlations among 32 non-m^6^A RNA modification regulators, which revealed that the crosstalk between the regulators of erasers, writers, and readers might participate in the formation of various non-m^6^A RNA modification patterns.

Next, we used ConsensusClusterPlus to sort patients with three non-m^6^A RNA modification patterns based on the expression profiles of 32 selected non-m^6^A RNA modification regulators. After unsupervised clustering, 144 cases were termed Cluster_A, 227 cases were identified in Cluster_B, and the other 168 cases were termed Cluster_C (**Figures 2B**, **S2B**). The expression of the 32 regulators in different clusters is shown in **Figure 2C**. In the survival analysis of non-m^6^A RNA modification patterns, a dominant survival disadvantage was found for the Cluster_B non-m^6^A RNA modification pattern (**Figure 2D**). To identify the biological functions of the three non-m^6^A RNA modification patterns, GSVA was performed. As exhibited in **Figure 2E**, Cluster_B was markedly enriched in pathways associated with the formation of the immunosuppressive TME and MES transition, such as extracellular matrix (ECM) receptor interaction, TGF b, JAK STAT, WNT, and NOTCH signal paths. In addition, Cluster_A was prominently enriched in pathways regulating the cell cycle, cell aging, and DNA damage, while Cluster_C was markedly related to apoptosis processes (**Figure S2C**).

### TME Cell Infiltration and MES Transition Characteristics in Distinct Non-m6A RNA Modification Patterns

**Figure 3:**
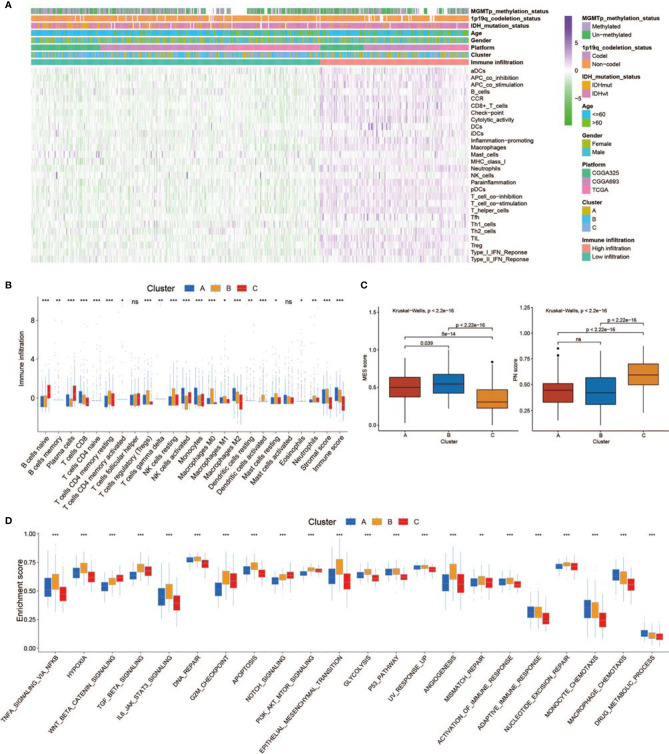
Tumor microenvironment (TME), subtypes, and clinical information in three non-m^6^A RNA modification clusters. **(A)** Unsupervised clustering of GBMs using ssGSEA scores from 28 immune-related gene sets. Two significant immune infiltration patterns are presented. The non-m^6^A RNA modification patterns and other clinical characteristics were used as patient annotations. **(B)** The abundance of tumor-infiltrating immune cells, stromal scores, and immune scores in three non-m^6^A RNA modification patterns. **(C)** Differences in mesenchymal/proneural (MES/PN) scores quantified by ssGSEA among the three non-m^6^A RNA modification patterns. **(D)** Differences in the TME infiltration and MES transition pathways among three non-m^6^A RNA modification patterns. * means P<0.05; ** means P<0.01; *** means P<0.001; ns means P>0.05.

SsGSEA was used to assess TME-infiltrating cells in GBM tissues. According to the abundance of infiltrating immune cells, we classified the GBM samples into high and low infiltration groups (**Figure 3A**). Patients with 1p19q codeletion status or IDH mutation status were mainly enriched in the low infiltration group (**Figure 3A**). Analyses of TME cell infiltration *via* the CIBERSORT algorithm confirmed that Cluster_B was obviously enriched in innate immune cell infiltration, including the infiltration of mast cells, natural killer (NK) cells, macrophages, and dendritic cells (DCs) (**Figure 3B**; **Table S2**). In addition, abundant stromal elements in the TME are associated with immunosuppression (71). We uncovered that the stromal score of Cluster_B was the highest among the three clusters *via* the ESTIMATE algorithm (**Figure 3B**). When we investigated the MES and PN scores, we found that the MES score was the highest and the PN score was the lowest in Cluster_B (**Figure 3C**). The MES-subtype GBM tended to exhibit an immunosuppressive TME. Previous studies demonstrated that there were abundant immune cells in tumors with an immune-excluded group, and immune cells participated in the formation of an immunosuppressive TME (72, 73). We found that three non-m^6^A patterns possessed definite TME infiltration characteristics. Cluster_B was termed an immune-excluded group, characterized by abundant innate immune cell infiltration, stromal activation, and a high MES score; Cluster_A was termed an immune-inflamed group, marked by abundant immune cell infiltration; and Cluster_C was termed an immune-desert phenotype, characterized by rare immune cell infiltration (**Figures 3A–C** and **S3A**). In addition, GBMs with an immune-excluded subtype had the worst prognosis (**Figure 2D**). Furthermore, to analyze the immune tolerance as well as activity condition of each pattern, we selected LGALS9, LAG3, ICOS, IL23A, LDHA, CTLA4, CD274, PTPRC, PDCD1, IL12A, ICOSLG, CD28, TNFRSF4, VTCN1, PDCD1LG2, TNFRSF18, TNFSF18, TNFSF4, CD80, CD86, CD8A, B2M, TNFSF9, YTHDF1, PVR, FGL1, CD40, SIGLEC15, LAMA3, CD40LG, HAVCR2, TNFRSF9, JAK2, JAK1, and LDHB as immune checkpoint-related molecules (74, 75). We uncovered that diverse immune checkpoint-related genes were significantly overexpressed in Cluster_B and Cluster_A (**Figure S3C**). In addition, we chose CD44, CHI3L1, FN1, SERPINE1, and TIMP1 as MES markers and OLIG2, DLL3, NCAM1, ASCL1, and SOX2 as PN markers (69, 76, 77). The expression of MES markers was high in Cluster_B, while the expression of PN markers was high in Cluster_C (**Figure S3D**). In addition, ssGSEA revealed that the immunosuppressive TME and MES transition pathways, such as the NF-κB and STAT3 signal paths, were obviously enriched in Cluster_B, whereas the monocyte and macrophage chemotaxis pathways were enriched in Cluster_A (**Figure 3D**). **Figure S3B** illustrates the proportion of GBM patients with the indicated clinical status and molecular traits in the three groups with different non-m^6^A RNA modification patterns. Patients over 60 years old and those who were alive at the last follow-up were primarily in Cluster_C, while patients with IDH wild-type status and no MGMT methylation were mainly found in Cluster_B (**Figure S3B**).

We analyzed the specific relationship between the TME-infiltrating cell type as well as non-m^6^A RNA modification regulators (**Figure S3E**). Patients were classified into low or high immune infiltration, MES, and PN score groups. Thirty-two non-m^6^A RNA modification regulator expression levels in different groups are shown in **Figures S3F–H**. We focused on UPP1, a U regulator, which had a significant positive correlation with numerous TME-infiltrating immune cells (**Figure S3E**). Patients were classified into low and high UPP1 expression subgroups, and a beneficial prognosis was presented in patients with low UPP1 (**Figure S4A**). We used ESTIMATE to assess the overall levels of immune cell infiltration as well as the stromal score. The results showed that patients with a high expression of UPP1 exhibited high immune and stromal scores (**Figure S4B**). The specific difference in TME-infiltrating levels between patients with low and high UPP1 expression was explored. We observed that tumors with high UPP1 showed obviously increased infiltration of Treg T cells and M2 macrophages (**Figure S4B**). We uncovered that increased UPP1 resulted in the overexpression of immune checkpoint-related genes (**Figure S4C**). In addition, the expression of MES markers was higher, while that of PN markers was lower in patients with high UPP1 expression (**Figure S4D**). Subsequent pathway enrichment analyses showed that high UPP1 samples exhibited an obvious enhancement of the immunosuppressive TME and MES transition-related pathways, including the NF-κB, STAT3, and epithelial–mesenchymal transition (EMT) signal paths (**Figure S4E**). From the above results, we speculated that UPP1-mediated U may promote the formation of an immunosuppressive TME and facilitate MES transition. In addition, we investigated the characteristics of one m^1^A and m^6^A_m_ eraser, FTO, which was negatively associated with various TME-infiltrating immune cells (**Figure S3E**). Patients with a high FTO exhibited a better prognosis (**Figure S5A**). We then found that a high expression of FTO was related to low immune and stromal scores (**Figure S5B**). We also discovered that a low FTO was related to the overexpression of a few immune checkpoint-related genes (**Figure S5C**). MES marker expression was higher, while PN marker expression was lower in patients with low FTO expression (**Figure S5D**). In addition, patients with low FTO expression showed prominent enrichment of the hypoxia pathway, STAT3 signaling pathway, and EMT signaling pathway (**Figure S5E**). Thus, FTO may inhibit the formation of an immunosuppressive TME and the process of MES transition.

**Figure 4:**
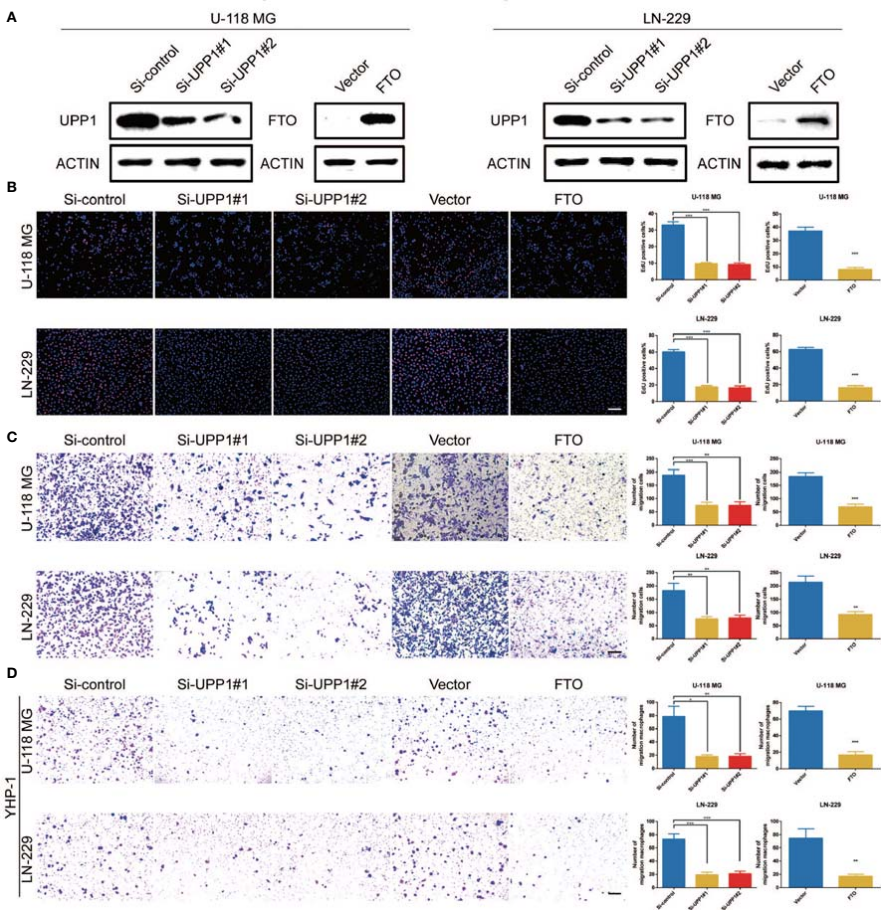
UPP1 knockdown and FTO overexpression inhibit cell proliferation, migration, and macrophage recruitment. **(A)** Western blot analysis was performed to assess the expression levels of UPP1 and FTO. ACTIN was used as a loading control. **(B)** EdU was performed in U-118 MG and LN-229 transfected si-control, si-UPP1#1, si-UPP1#2, vector, and ov-FTO cells (scale bar = 100 μm). **(C)** Transwell assay performed in U-118 MG and LN-229 transfected si-control, si-UPP1#1, si-UPP1#2, vector, and ov-FTO cells (scale bar = 100 µm). **(D)** Transwell assays showed the ability of growth media of different GBM cells to recruit macrophages (scale bar = 100 µm). * means P<0.05; ** means P<0.01; *** means P<0.001.

To assess the role of UPP1 and FTO in diverse cellular processes of GBM cells, several experiments were performed. Downregulation of UPP1 or overexpression of FTO resulted in a significant decrease in the EdU-positive cell percentage (**Figures 4A, B**). In addition, the results of the Transwell assays revealed that UPP1 silencing and FTO overexpression reduced the number of GBM cells migrating to the membrane (**Figure 4C**). Interestingly, we found that UPP1 knockdown or FTO overexpression significantly inhibited the capability of conditioned medium from GBM cells to recruit THP1-differentiated macrophage (**Figure 4D**).

### Non-m^6^A RNA Modification Phenotype-Related DEGs in GBM

**Figure 5:**
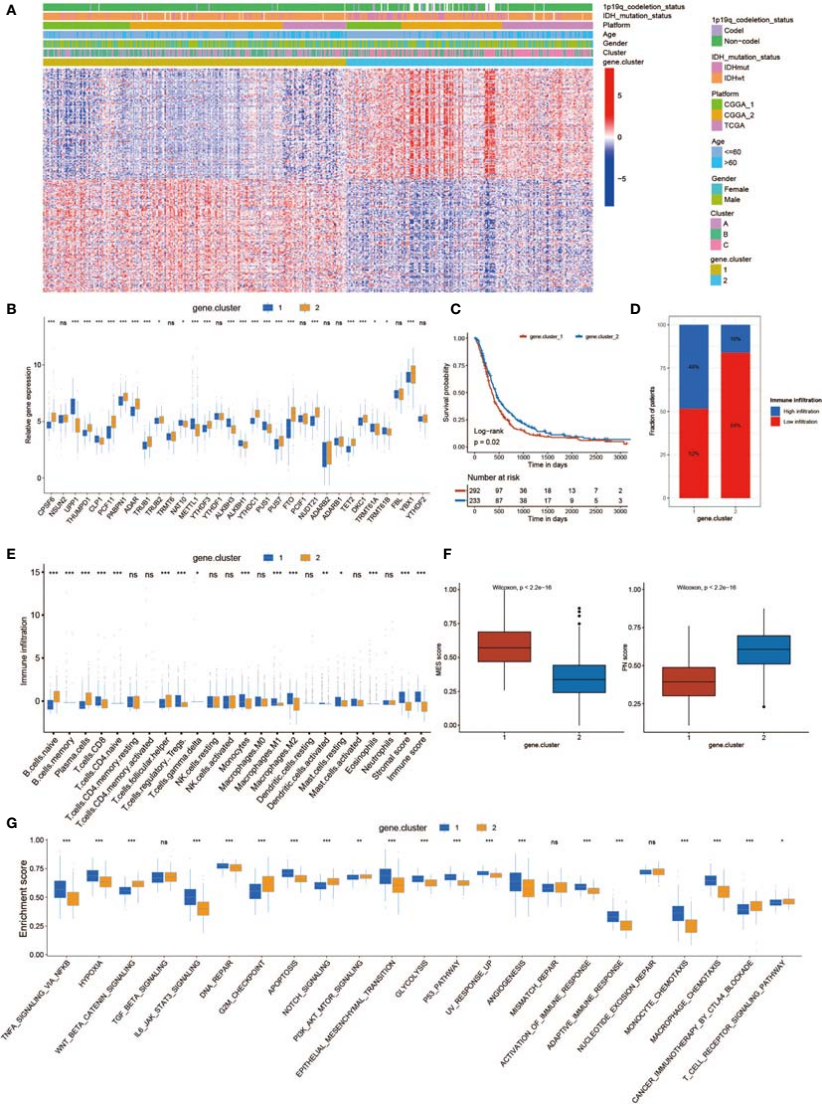
Generation of a non-m^6^A RNA modification-related gene set. **(A)** Unsupervised clustering of 150 DEGs dividing patients into two gene.clusters, named gene.cluster_1 and gene.cluster_2. Cluster, gender, age, platform, IDH status, and 1p19q status were applied for sample annotation. **(B)** The expression of 32 non-m^6^A RNA modification regulators in two gene.clusters. **(C)** Survival analyses for the gene.cluster_1 (292 cases) and gene.cluster_2 (233 cases) cohorts (*p* = 0.02, log-rank test). **(D)** The proportion of immune infiltration in the two gene.clusters. **(E)** The abundance of each TME-infiltrating cell, stromal scores, and immune scores in the two gene.clusters. **(F)** Differences in MES/PN scores among the two gene.clusters. **(G)** Differences in the TME infiltration and MES transition pathways between the two gene.clusters. * means P<0.05; ** means P<0.01; *** means P<0.001; ns means P>0.05.

To characterize the potential biological behavior of the three non-m^6^A patterns, we confirmed 243 RNA phenotype-related DEGs (**Figure S6A**). Then, a univariate Cox regression model was applied to perform prognostic analysis for each DEG in the signature (**Table S3**). The DEGs with significant prognostic value were extracted for further analysis. To further corroborate this regulatory mechanism, unsupervised clustering methods were executed according to 150 DEGs (**Figure S6B**). The marked enriched biological functions of these DEGs are presented in **Figure S6C**. The unsupervised clustering algorithm sorted the cases into two subtypes: gene.cluster_1 and gene.cluster_2 (**Figure 5A**). The two patterns were identified in gene.cluster_1 and gene.cluster_2, marked by different signature genes (**Figure 5A**). Patients with 1p19q codeletion status or IDH mutation status were mainly concentrated in gene.cluster_2 (**Figure 5A**). In addition, in the two non-m^6^A RNA modification DEG clusters, noticeable differences in non-m^6^A RNA modification regulator expression were analyzed (**Figure 5B**). A total of 242 patients with GBM were termed gene.cluster_2, which was validated to be correlated with a favorable prognosis (**Figure 5C**). To our surprise, gene.cluster_1 was remarkably abundant in several immune cell infiltrates (**Figures 5D, E**). In addition, the immune cell infiltration of gene.cluster_1 was stronger than that of gene.cluster_2 (**Figures 5D, E**). However, we found that the stromal score of gene.cluster_1 was higher than that of gene.cluster_2 (**Figure 5E**). In addition, gene.cluster_1 possessed a higher MES score and lower PN score than gene.cluster_2 (**Figure 5F**). These results indicate that patients in gene.cluster_1 had a highly immunosuppressive TME, which is related to a poor prognosis. Moreover, we determined that diverse immune checkpoint-related genes were significantly overexpressed in gene.cluster_1 (**Figure S6D**). In addition, the expression of MES markers was high in gene.cluster_1, while the expression of PN markers was high in gene.cluster_2 (**Figure S6E**). Moreover, ssGSEA revealed that the immunosuppressive TME and MES pathways were significantly enriched in gene.cluster_1, whereas T-cell receptor signaling pathways were enriched in gene.cluster_2 (**Figure 5G**).

### Generation of the RM Score and Analysis of Clinical Traits

**Figure 6:**
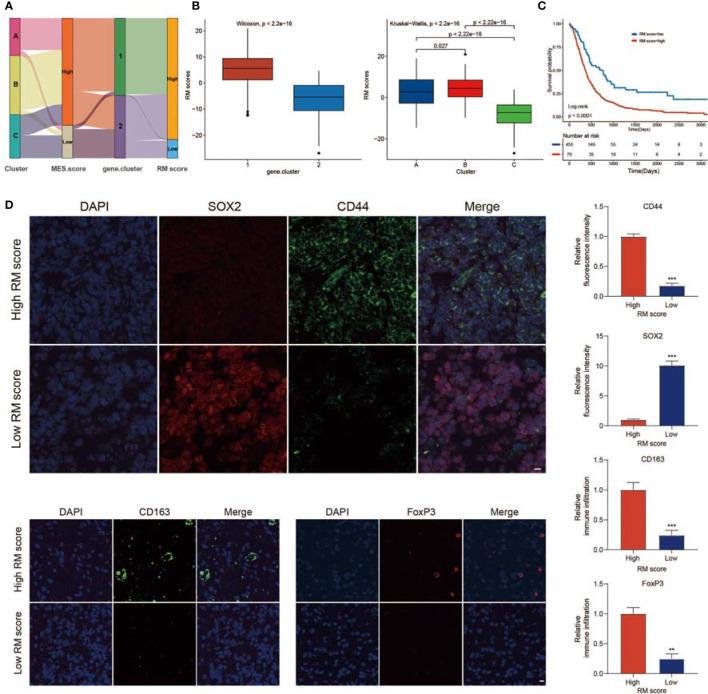
Calculation of regulator score (RM) score. **(A)** Sankey plot showing the changes in non-m^6^A RNA modification patterns, MES scores, gene.clusters, and RM scores. **(B)** Differences in RM scores among the two gene.clusters and three non-m^6^A RNA modification patterns. **(C)** Survival analyses for the high RM score and low RM score patient groups. **(D)** IF staining in GBM tissues showed the expression of CD44, SOX2, CD163, and FoxP3. Histogram representing relative fluorescence intensity (scale bar = 15 μm). ** means P<0.01; *** means P<0.001.

The above results showed that non-m^6^A plays an essential role in shaping different TME landscapes as well as facilitating MES transition. However, these analyses only assessed the characteristics of the population and could not correctly evaluate the detailed information of non-m^6^A RNA modification of each GBM case. Taking into account the complexity and individual heterogeneity of non-m^6^A RNA modifications, we generated a scoring model to assess the non-m6A RNA modification patterns of individual GBM patients according to phenotype-related DEGs; this system was termed the RM score. The relationships among the non-m^6^A RNA modification patterns, MES score, gene.cluster, and RM score were visualized in an alluvial diagram and in **Table S4** (**Figure 6A**). The Wilcoxon test revealed an obvious difference in the RM score between the gene.clusters. Gene.cluster_2 showed a lower median score than gene.cluster_1, which indicated that a high RM score could be closely linked to the immunosuppressive TME and MES transition signatures (**Figure 6B**). Moreover, Cluster_B showed a significantly increased RM score compared with Cluster_A and Cluster_C (**Figure 6B**). In addition, patients with stronger immune cell infiltration possessed higher RM scores (**Figure S7A**).

We investigated the value of the immune, MES, and PN scores in predicting patient prognosis. A high immune score, high MES score, or low PN score indicated a poor prognosis (**Figure S6F**). In addition, we observed a significantly negative relationship between the MES score and PN score (**Figure S6G**). Next, we evaluated the significance of the RM score in predicting GBM outcomes. With a cutoff value of −10.21, GBMs were classified into high or low RM score groups. GBMs with lower RM scores presented a significantly prolonged survival time (**Figure 6C**). To evaluate the stability of the RM scoring system, we used the RM score in other independent GBM databases to verify its prognostic significance, and the results indicated that a low RM score was correlated with better clinical benefit (**Figures S8A, B**). We analyzed whether the RM score could be regarded as an independent factor of survival for GBM. Multivariate Cox regression analysis was performed, confirming that the RM score was an independent and robust factor for predicting GBM patient outcomes [**Figure S8C**; HR (low RM score) = 0.53 (0.39–0.73)]. We also examined the survival prediction efficiency for the combination of RM score with immune score, RM score and MES score, and RM score and PN score. The results presented that the low RM and low immune score groups showed the best OS among the groups (**Figure S7B**). Furthermore, we observed that patients with low RM and MES scores or low RM scores and high PN scores had the most significant survival benefits (**Figures S8D, E**). We assessed the value of the RM scoring feature to evaluate the efficacy of radiotherapy or chemotherapy in GBMs. Among the patients who received adjuvant radiotherapy or chemotherapy at the same time, those with low RM scores exhibited the most significant therapeutic benefit (**Figure S7C**). Furthermore, patients over 60 years old, patients alive at the last follow-up, and patients with IDH mutations, 1p/19q codeletion, and MGMT methylation were mainly found in the low RM score group (**Figures S8F, G**).

Further analysis showed that the immune cell infiltration of patients with high RM scores was significantly higher than that of patients with low RM scores, while patients with high RM scores had higher stromal scores (**Figure S7D**). In addition, patients with high RM scores had higher MES scores and lower PN scores (**Figure S7E**). GBM patients with the MES subtype were characterized by higher TME levels, whereas GBM patients with the PN subtype were associated with lower TME levels. To exclude the influence of phenotypic and tumor immune infiltration correlations on the analysis results, we analyzed the correlation between RM scores and TME levels in patients with PN and MES subtypes (**Figure S9**). Moreover, to reveal the significance of the RM score in estimating the level of TME immune infiltration and MES transition, we studied the expression of immune checkpoint-related genes and MES/PN marker genes in different RM score groups. We found that most immune checkpoint-related genes were upregulated in the high RM score group (**Figure S7F**). In addition, the expression of MES markers was higher in high RM score patients, while the expression of PN markers was higher in low RM score patients (**Figure S7G**). We examined the relationship between the RM score and known biological signatures using Spearman analysis. A heatmap of the association matrix exhibited that the RM score was positively related to immunosuppressive TME and MES transition signatures (**Figure S7H**).

To verify the role of the RM score in evaluating the characteristics of TME infiltration and MES transition, IF staining was performed. The proportions of M2 macrophages (CD163^+^) and FoxP3^+^ Tregs infiltrated in tumors with high RM scores were higher than those in tumors with low RM scores (**Figure 6D**). Moreover, CD44 expression was also markedly increased in the high RM score group, while SOX2 expression was decreased (**Figure 6D**). These results further validated that tumors with high RM scores showed an immunosuppressive phenotype.

### The Correlation Between the RM Score and TMB

**Figure 7:**
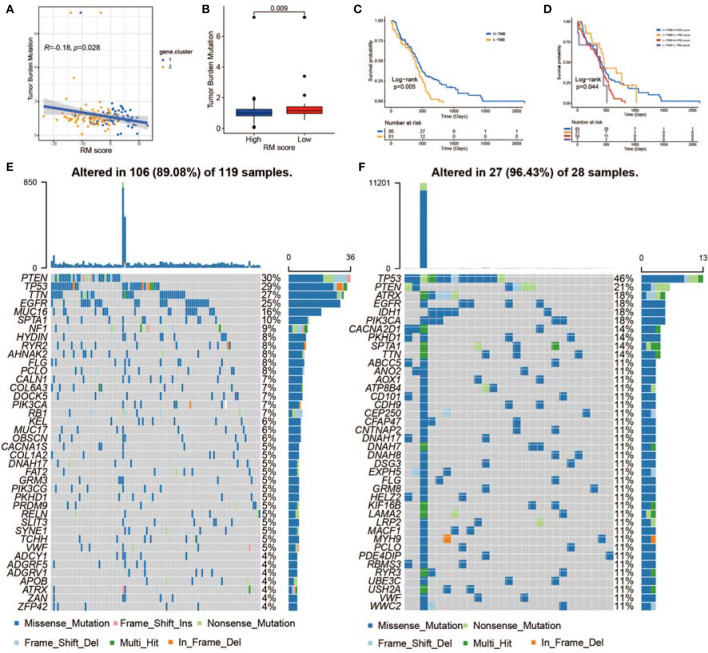
The correlation between the RM score and TME infiltration, MES transition, and somatic variants. **(A)** Scatterplots indicating the negative relationship between RM score and TMB in TCGA-GBMs (*r* = −0.18, *p* < 0.028, Spearman correlation analysis). **(B)** TMB difference in the low and high RM score cohorts. **(C)** KM curves for the low and high TMB GBMs. H, high; L, low. **(D)** Survival analyses for subgroup GBMs stratified by RM score and TMB. **(E, F)** Waterfall plot showing tumor somatic mutations presented by those with high RM scores **(E)** and low RM scores **(F)**.

Increasing evidence has demonstrated a significant correlation between responsiveness to immunotherapy and tumor mutation burden (TMB) (78). Taking the clinical significance of TMB into account, we further analyzed the relationship between the RM score and the TMB. By comparing the TMB values for patients with high and low RM scores, we concluded that the RM score was negatively associated with the TMB value (**Figure 7A**). Patients with higher RM scores exhibited a lower TMB than patients with lower RM scores (**Figure 7B**). We divided the patients into different groups according to the immune setting of the TMB, as described previously (79). As shown in **Figure 7C**, we observed that patients with a high TMB had prolonged survival times compared with those with a low TMB. Considering the contrary survival significance of the TMB and RM score, we analyzed the cross-influence of these scores on the prognosis of patients with GBM. Stratified survival analyses revealed that patients with low RM scores together with high TMB scores had the most significant survival benefits (**Figure 7D**). Furthermore, we evaluated the somatic variant distribution of GBM driver genes among the high and low RM score groups using the maftools package. As shown in **Figures 7E, F**, the low RM score group presented more extensive TMB than the high RM score group. An increasing number of studies have shown that patients with a high TMB are more sensitive to anti-PD-1/PD-L1 immunotherapy (80). In addition, the alteration frequency of various genes was different between the low and high RM score groups (**Figures 7E, F**). In conclusion, the above findings corroborated that the difference in non-m^6^A RNA modification patterns could be an important factor that predicted response efficiency to anti-PD-1/PD-L1 immunotherapy. These results might lead to novel strategies for exploring the mechanism of non-m^6^A RNA modification composition and gene mutation in ICB therapy.

### The Predictive Value of the RM Score in Predicting ICB Therapy Response

**Figure 8:**
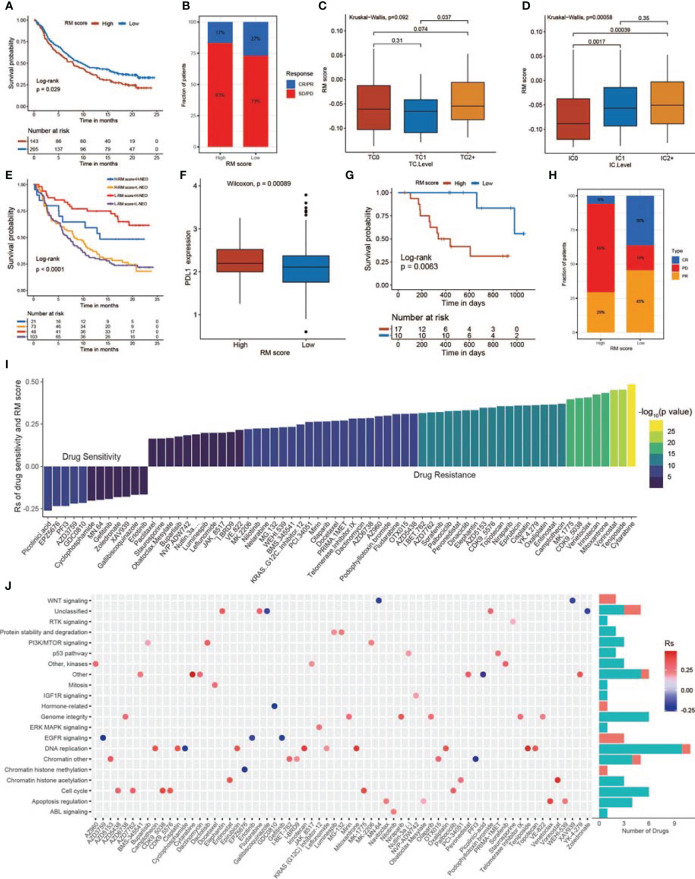
The relationship between RM score and response to immunotherapy and drug sensitivity. **(A)** Survival analysis of the low and high RM score patient groups in an immune checkpoint blockade (ICB) therapy cohort. **(B)** The proportion of patients who responded to ICB therapy in the low or high RM score groups. **(C)** Differences in RM scores among the three tumor cell (TC) levels. (TC levels, level of IHC-assessed PD-L1 staining of tumor cells. TC0, <1%; TC1, ≥1% but <5%; TC2^+^, ≥5% of tumor cells with staining for PD-L1, *p* = 0.092). **(D)** Differences in RM scores among the three immune cell (IC) levels. (IC levels, level of IHC-assessed PD-L1 staining of immune cells. IC0, <1%; IC1, ≥1% but <5%; IC2^+^, ≥5% of immune cells staining for PD-L1, *p* = 0.00058). **(E)** Survival analyses of patients receiving ICB therapy stratified by RM score and TMB. **(F)** Differences in PD-L1 expression between the high and low RM score patients. **(G)** Survival analyses of the high and low RM score patient groups in another ICB therapy cohort (GSE78220). **(H)** Proportion of patients who responded to ICB therapy in the low or high RM score groups. **(I)** The relationship between RM score and drug AUC value. **(J)** Signal paths targeted by drugs whose AUC values are positive (red) or negative (blue) with RM score.

Significant efforts have been made to screen biomarkers that predict ICB treatment response; some previously studied biomarkers include the TMB as well as the PD-L1 expression level (81). Considering that the RM score is related to the TME, we examined the value of the RM score in predicting the response of GBMs to immunotherapy. The exploration was based on two independent immunotherapy studies (**Figures 8A–H**). We observed that patients who had lower RM scores exhibited obviously prolonged OS in the anti-PD-L1 research (IMvigor210; **Figure 8A**) and anti-PD-1 research (GSE78220; **Figure 8G**) (74, 82). The patients in the IMvigor210 cohort exhibited diverse degrees of response to anti-PD-L1 blockers, including partial response (PR), progressive disease (PD), complete response (CR), and stable disease (SD). We explored the differences in RM scores for patients with diverse responses to ICB therapy and concluded that the proportion of patients in the response groups (CR and PR) was significantly lower in the high RM score group than in the low RM score group, while the proportion of patients in the no/limited response groups (SD and PD) showed the opposite trend, indicating that the RM score could reveal the response of patients to ICB therapy (**Figure 8B**). The expression of PD-L1 on tumor cells (TCs) was substantially related to the RM score. The TC1 group exhibited the lowest RM score and was obviously different from the TC0 as well as TC2^+^ groups (**Figure 8C**). Moreover, we observed that the PD-L1 level of immune cells (ICs) was positively related to the RM score, with IC0 exhibiting the lowest RM score and IC2 exhibiting the highest RM score (**Figure 8D**). By analyzing the relationship of the RM score with tumor neoantigen burden, we observed that patients with low RM scores together with a high neoantigen burden exhibited the most prolonged survival time (**Figure 8E**). In addition, patients with high RM scores showed significantly high levels of PD-L1 (**Figure 8F**). Finally, we confirmed the significant clinical response and therapeutic advantages of anti-PD-1 immunotherapy in patients with low RM scores versus those with high RM scores (**Figures 8H**, **S8H**). The RM score was also negatively correlated with the TMB in the GSE78220 cohort (**Figure S8I**).

### Exploration of the Therapeutic Significance of the RM Score

To investigate the value of the RM score in predicting drug sensitivity, we calculated the correlation between the RM score and the AUC for drugs in multiple cancer cell lines. According to Spearman correlation analysis, we selected 68 drugs for which the RM score and drug sensitivity were significantly correlated in the GDSC database (**Figure 8I**) (83). The RM score was positively correlated with sensitivity to 12 drugs, including picolinic acid, chromatin histone methylation inhibitor EPZ5676, EGFR inhibitor AZD3759, gefitinib, erlotinib, and WNT inhibitors MN.64 and XAV939; the RM score was negatively correlated with drug sensitivity (and this positively correlated with drug resistance) for 56 drugs (**Figures 8I, J**). Furthermore, we explored the signal path targeted by the selected drugs. We observed that drugs whose sensitivity was positively related to the RM score targeted the EGFR and WNT signaling pathways. In contrast, drugs whose sensitivity was negatively related to the RM score targeted the cell cycle and apoptosis signal path (**Figure 8J**). In conclusion, these results revealed that the RM score model might serve as a potential factor for exploring proper therapy strategies.

### Construction of the m^6^A Score and Comparison of the m^6^A and RM Scores

**Figure 9:**
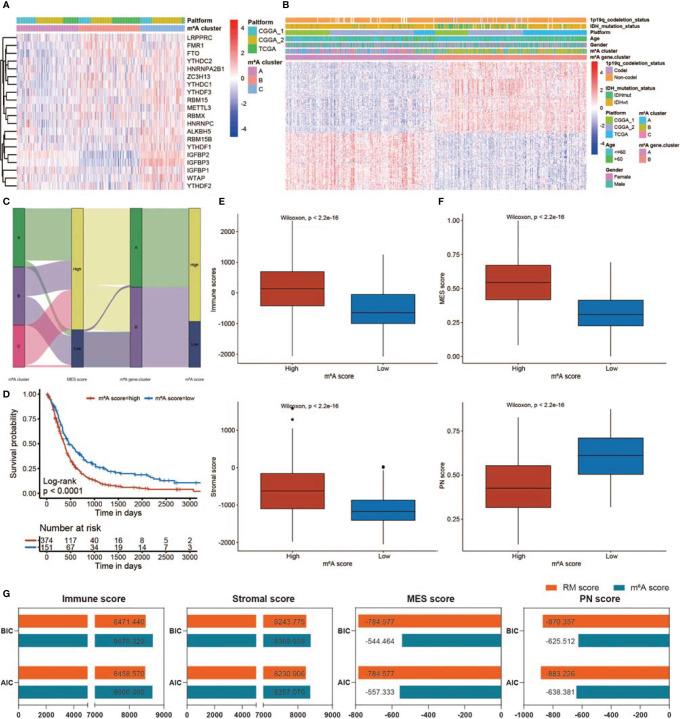
Construction of the m^6^A score and comparison of the m^6^A and RM scores. **(A)** Heatmap showing unsupervised clustering for 20 m^6^A RNA modification genes in 539 GBM patients. Each column represents patients, and each row represents an m^6^A RNA modification regulator. **(B)** Unsupervised clustering of 172 DEGs dividing patients into two m^6^A gene.clusters, termed m^6^A gene.cluster_1 and m^6^A gene.cluster_2. The m^6^A gene.cluster, m^6^A cluster, sex, age, platform, IDH status, and 1p19q status were applied for patient annotation. **(C)** Sankey diagram showing the distribution of m^6^A RNA modification patterns, MES scores, m^6^A gene.clusters, and m^6^A scores. **(D)** KM curve showing survival analysis results of the high m^6^A score and low m^6^A score patient groups. **(E)** Differences in immune/stromal scores among GBMs with low m^6^A scores and high m^6^A scores. **(F)** Differences in MES/PN scores among GBMs with low m^6^A scores and high m^6^A scores. **(G)** Comparison of RM and m^6^A scores using BIC and AIC values.

A total of 20 m^6^A RNA modification regulators were enrolled in this study. Next, we classified patients into different m^6^A RNA modification patterns according to the expression profiles of 20 selected m^6^A RNA modification genes. After unsupervised clustering, 199 cases were termed m^6^A Cluster_A, 197 cases were termed m^6^A Cluster_B, and the other 143 cases were identified in m^6^A Cluster_C (**Figure 9A**). In the survival analysis of m^6^A RNA patterns, m^6^A Cluster_B exhibited a particularly outstanding survival benefit (**Figure S10A**; *p* = 0.0018). In addition, we identified 295 DEGs using the limma package. Then, univariate Cox regression analysis was applied to determine the prognostic value for each DEG (**Table S5**), and the DEGs with significant prognostic value were extracted for further analysis. To further corroborate this regulatory information, an unsupervised clustering method was used according to the 172 DEGs. The patients were divided into two groups, m^6^A gene.cluster_1 and m^6^A gene.cluster_2, using the unsupervised clustering algorithm (**Figure 9B**). A total of 272 patients with GBM were termed m^6^A gene.cluster_2, which was validated to be associated with a better prognosis (**Figure S10B**). Based on these DEGs, we generated a scoring system to assess the m^6^A RNA modification pattern of each GBM patient. The relationships among m^6^A RNA modification patterns, MES score, m^6^A gene.cluster, and m^6^A score were visualized in an alluvial diagram and in **Table S6** (**Figure 9C**). Patients with low m^6^A scores demonstrated a prolonged survival time (**Figure 9D**). The Wilcoxon test revealed a significant difference in the m^6^A score between the m^6^A gene.clusters. M^6^A gene.cluster_2 showed a lower median score than gene.cluster_1, which indicated that a high m^6^A score could be closely linked to poor prognosis (**Figure S10C**). Moreover, compared with the other clusters, m^6^A Cluster_B showed a significantly decreased m^6^A score (**Figure S10D**). In addition, patients with high RM scores exhibited higher immune, stromal, and MES scores and lower PN scores (**Figures 9E, F**).

The value of the RM and m^6^A scores in predicting patient outcomes and the TME was compared using the BIC and AIC values. With lower BIC and AIC values, the RM scoring model exhibited a better description of TME infiltration and MES transition than the m^6^A scoring model (**Figure 9G**). There was no obvious difference between the RM and m^6^A scores in predicting prognosis (**Figure S10E**).

### RM Score Correlates With Immune Infiltration and Survival Prognosis in Pan-Cancer

According to the forest plots, a positive association was obvious between RM score and OS in KIRC, LGG, and LAML (**Figure S11A**). In addition, the PFS forest plot confirmed the role of RM score as a risk factor in KIRC and LGG (**Figure S11A**). As presented in **Figure S11B**, RM score is positively related to the TMB in BRCA, KIRC, LIHC, and THCA, whereas a negative association was observed in LAML and THYM. For microsatellite instability (MSI), a positive association in BRCA, DLBC, HNSC, PRAD, SKCM, and THCA, as well as a negative association in ACC, CESC, COAD, KUAD, LUSC, and UCEC, was identified (**Figure S11B**). In terms of immune cell infiltration, RM score was positively associated with regulatory T-cell content in BRCA, COAD, KIRC, KIRP, LGG, SKCM, UCEC, and UCS (**Figure S11C**). In BLCA, CESC, KICH, LAML, and TGCT, RM score was positively associated with M2 macrophage infiltration (**Figure S11C**).

## Discussion

Increasing evidence has demonstrated that various RNA epigenetic modifications have an important effect on infection, the TME, and antitumor proliferation by interacting with several regulators. However, most studies have focused on a single regulator or a single kind of RNA modification, such as m^6^A, and the mutual relationships and effects of regulators of various non-m^6^A RNA modifications in cancer are not fully understood. Few studies have demonstrated that a given non-m^6^A RNA modification influences tumorigenesis in GBM, probably because non-m^6^A RNA modifications are not as abundant as m^6^A modifications.

Here, we revealed global non-m^6^A RNA alterations at the genetic as well as transcriptional levels and showed mutual correlations in GBMs. Surprisingly, there are complicated associations among 32 non-m^6^A RNA alteration regulators. We constructed three non-m^6^A RNA modification patterns based on 32 regulators. Cluster_A was characterized by abundant immune cell enrichment and classified as an immune-inflamed subtype; Cluster_B was characterized by the suppression of immune function as well as MES transition, corresponding to the immune-excluded subtype; and Cluster_C was marked by rare immune cell infiltration, corresponding to the immune-desert subtype. Although the immune-excluded subtype was related to abundant immune cell infiltration, the immune cells were inactive because of the distribution of effective immune cells in the tumor stroma. The stroma could permeate the tumor itself or might be confined to the tumor envelope, making immune cells appear to be inside the tumor (84). In addition, the MES score was the highest and the PN score was the lowest in Cluster_B. The immunosuppressive TME and MES transition pathways, such as the NF-κB and STAT3 signaling pathway, were markedly enriched in Cluster_B. The MES-subtype GBM tends to exhibit an immunosuppressive TME, while an immunosuppressive TME promotes GBM malignant progression *via* MES transition (62, 64). Our data demonstrated that there may be a positive feedback loop between the immunosuppressive TME and MES transition, resulting in a poor prognosis.

Furthermore, the mRNA expression differences among non-m^6^A RNA modification patterns have been confirmed to be significantly related to RNA modification-, immune-, and MES-related biological signaling pathways. The DEGs were considered a non-m^6^A RNA modification-related gene set. Similarly, two non-m^6^A-related gene subtypes were identified based on the DEGs, which were obviously related to the immunosuppressive TME as well as MES transition signaling pathways. The MES subtype is particularly aggressive among these three subtypes, while the PN subtype has the best prognosis (85). This demonstrated again that the various non-m^6^A RNA modifications were of great significance in the formation of TME patterns. In conclusion, a systematic evaluation of the non-m^6^A RNA modification patterns will improve our understanding of TME cell infiltration and MES transition characterization.

Considering the heterogeneity of non-m^6^A RNA modifications across individual tumors, a method for quantifying the non-m^6^A RNA modification patterns of individual tumors is urgently needed. Accordingly, we developed a scoring model, the RM score, to evaluate the non-m^6^A RNA modification pattern of individual GBMs. Patients with low RM scores demonstrated a prominent survival benefit. Patients in Cluster_B, characterized by an immune-excluded phenotype, exhibited a higher RM score. In addition, the RM score was significantly positively associated with immunosuppressive TME and MES transition signatures.

Immunotherapy is a developing field but is far from reaching clinical expectations in GBM patients because of the immunosuppressive TME. The MES transition not only promotes radiochemotherapy resistance but also shapes the immunosuppressive TME in GBM. Numerous studies have emphasized the non-negligible interaction between non-m^6^A RNA regulators, the TME, and the MES transition. The RM scoring model could predict patient prognosis and sensitivity to radiochemotherapy as well as immunotherapy. Patients who were sensitive to radiochemotherapy and ICB therapy were mainly enriched in the low RM score group. In addition, the RM score was markedly positively correlated with immunosuppressive TME and MES transition signatures.

Our findings also revealed a significantly negative association between the RM score and TMB. Increasing evidence has demonstrated that patients with a high TMB present acceptable responses to immunotherapy. A high TMB status was correlated with a favorable prognosis. In addition, RM score could predict the response of a patient to anti-PD-1/L1 immunotherapy. Patients receiving ICB therapy in the IMvigor210 and GSE78220 cohorts were assessed, and we identified that the RM score was markedly decreased in patients responding to ICB, which validated the predictive significance of the RM score model. Overall, this study showed that patients with low RM scores had more obvious benefits from immunotherapy.

In addition, we investigated the possible treatment outcome of non-m^6^A RNA modification regulators in GBMs. The RM score was positively correlated with the resistance of drugs that targeted the cell cycle and apoptosis signaling pathways and negatively correlated with the sensitivity of drugs that targeted the EGFR and WNT signaling pathways. These findings indicated that patients with higher RM scores might be more suitable for drugs targeting the EGFR and WNT signal paths instead of drugs targeting the cell cycle or apoptosis signaling pathways. Thus, non-m^6^A RNA modification patterns could be used as a qualified predictor that can be used to predict the clinical outcome of targeted therapies or chemotherapy. Our findings reveal novel possibilities for improving the efficacy of ICB treatment, revealing different TME phenotypes as well as MES subtypes and suggesting the potential for personalized and precise immunotherapy for GBM.

Increasing evidence has demonstrated that the m^6^A modification pattern is closely associated with the TME, prognosis, and other clinical characteristics. We constructed an RM scoring system to evaluate the potential roles of RNA modifications beyond m^6^A in the TME, MES transition, immunotherapy, and drug sensitivity. To further evaluate the superiority of the RM scoring model compared with the m^6^A modification pattern, we constructed an m^6^A scoring model and compared two models using the BIC and AIC algorithms. Finally, we confirmed that the RM scoring system is better than the m^6^A scoring system in assessing TME and GBM subtypes.

In this study, we systematically assessed the non-m^6^A RNA modification patterns of 539 GBMs on the basis of 32 regulator genes and associated these patterns with TME infiltration as well as MES transition characteristics. We constructed the RM score model to predict patient prognosis and the response to immunotherapy and targeted therapy. The systematic assessment of non-m^6^A RNA modification patterns could not only improve our knowledge of the crosstalk of RNA modifications but also contribute to the development of more personalized and precise immunotherapy regimens.

## Data Availability Statement

Publicly available datasets and online tools were applied in this study. These resources could be found here: TCGA: https://portal.gdc.cancer.gov/; https://xena.ucsc.edu/; CGGA: http://www.cgga.org.cn/; GEO: https://www.ncbi.nlm.nih.gov/geo/query/acc.cgi?acc=GSE78220; GDSC: https://www.cancerrxgene.org/; CCLE: https://portals.broadinstitute.org/ccle/; IMvigor210: http://research-pub.gene.com/IMvigor210CoreBiologies/packageVersions.

## Ethics Statement

The studies involving human participants were reviewed and approved by the Ethics Committee of the Qilu Hospital (Jinan, China) and performed in accordance with the relevant guidelines and regulations; the patient data were acquired from the publicly available datasets, and informed consent was given.

## Author Contributions

JX, ZG, and GL designed this work. JX, ZG, HX, and XG integrated and analyzed the data. JX, ZG, KL, ZZ, PZ, and LD wrote this manuscript. JX, ZG, YF, SW, HW, QW, and RZ edited and revised the manuscript. All authors contributed to the article and approved the submitted version.

## Funding

This work was supported by grants from the National Natural Science Foundation of China (Nos. 81874083; 82072776; 82072775; 81702468; 81802966; 81902540, 81874082; 81472353), Natural Science Foundation of Shandong Province of China (Nos. ZR2019BH057; ZR2020QH174; ZR2021LSW025), the Jinan Science and Technology Bureau of Shandong Province (2021GXRC029), Key clinical Research project of Clinical Research Center of Shandong University (2020SDUCRCA011) and Taishan Pandeng Scholar Program of Shandong Province (No. tspd20210322).

## Conflict of Interest

The authors declare that the research was conducted in the absence of any commercial or financial relationships that could be construed as a potential conflict of interest.

## Publisher’s Note

All claims expressed in this article are solely those of the authors and do not necessarily represent those of their affiliated organizations, or those of the publisher, the editors and the reviewers. Any product that may be evaluated in this article, or claim that may be made by its manufacturer, is not guaranteed or endorsed by the publisher.

## References

[1] Lapointe S Perry A Butowski NA. Primary Brain Tumours in Adults. Lancet (2018) 392(10145):432–46. doi: 10.1016/S0140-6736(18)30990-5 30060998

[2] Yang F He Z Duan H Zhang D Li J Yang H. Synergistic Immunotherapy of Glioblastoma by Dual Targeting of IL-6 and CD40. Nat Commun (2021) 12(1):3424. doi: 10.1038/s41467-021-23832-3 34103524 PMC8187342

[3] Wang Q Hu B Hu X Kim H Squatrito M Scarpace L. Tumor Evolution of Glioma-Intrinsic Gene Expression Subtypes Associates With Immunological Changes in the Microenvironment. Cancer Cell (2017) 32(1):42–56.e6. doi: 10.1016/j.ccell.2017.06.003 28697342 PMC5599156

[4] Zhang N Zhang H Wang Z Dai Z Zhang X Cheng Q. Immune Infiltrating Cells-Derived Risk Signature Based on Large-Scale Analysis Defines Immune Landscape and Predicts Immunotherapy Responses in Glioma Tumor Microenvironment. Front Immunol (2021) 12(3149). doi: 10.3389/fimmu.2021.691811 PMC8418124 34489938

[5] Boccaletto P Machnicka MA Purta E Piatkowski P Baginski B Wirecki TK. MODOMICS: A Database of RNA Modification Pathways. 2017 Update. Nucleic Acids Res (2018) 46(D1):D303–D7. doi: 10.1093/nar/gkx1030 PMC5753262 29106616

[6] Chen K Song B Tang Y Wei Z Xu Q Su J. Rmdisease: A Database of Genetic Variants That Affect RNA Modifications, With Implications for Epitranscriptome Pathogenesis. Nucleic Acids Res (2021) 49(D1):D1396–D404. doi: 10.1093/nar/gkaa790 PMC7778951 33010174

[7] Cheray M Etcheverry A Jacques C Pacaud R Bougras-Cartron G Aubry M. Cytosine Methylation of Mature Micrornas Inhibits Their Functions and Is Associated With Poor Prognosis in Glioblastoma Multiforme. Mol Cancer (2020) 19(1):36. doi: 10.1186/s12943-020-01155-z 32098627 PMC7041276

[8] Dominissini D Moshitch-Moshkovitz S Schwartz S Salmon-Divon M Ungar L Osenberg S. Topology of the Human and Mouse M6a RNA Methylomes Revealed by M6a-Seq. Nature (2012) 485(7397):201–6. doi: 10.1038/nature11112 22575960

[9] Zhao BS Roundtree IA He C. Post-Transcriptional Gene Regulation by Mrna Modifications. Nat Rev Mol Cell Biol (2017) 18(1):31–42. doi: 10.1038/nrm.2016.132 27808276 PMC5167638

[10] Zhao BS Wang X Beadell AV Lu Z Shi H Kuuspalu A. M(6)a-Dependent Maternal Mrna Clearance Facilitates Zebrafish Maternal-to-Zygotic Transition. Nature (2017) 542(7642):475–8. doi: 10.1038/nature21355 PMC5323276 28192787

[11] Yoon KJ Ringeling FR Vissers C Jacob F Pokrass M Jimenez-Cyrus D. Temporal Control of Mammalian Cortical Neurogenesis by M(6)a Methylation. Cell (2017) 171(4):877–89.e17. doi: 10.1016/j.cell.2017.09.003 28965759 PMC5679435

[12] Zhang C Chen Y Sun B Wang L Yang Y Ma D. M(6)a Modulates Haematopoietic Stem and Progenitor Cell Specification. Nature (2017) 549(7671):273–6. doi: 10.1038/nature23883 28869969

[13] Barbieri I Tzelepis K Pandolfini L Shi J Millan-Zambrano G Robson SC. Promoter-Bound METTL3 Maintains Myeloid Leukaemia by M(6)a-Dependent Translation Control. Nature (2017) 552(7683):126–31. doi: 10.1038/nature24678 PMC6217924 29186125

[14] Wiener D Schwartz S. The Epitranscriptome Beyond M(6)a. Nat Rev Genet (2021) 22(2):119–31. doi: 10.1038/s41576-020-00295-8 33188361

[15] Shi H Chai P Jia R Fan X. Novel Insight Into the Regulatory Roles of Diverse RNA Modifications: Re-Defining the Bridge Between Transcription and Translation. Mol Cancer (2020) 19(1):78. doi: 10.1186/s12943-020-01194-6 32303268 PMC7164178

[16] Zhao Z Meng J Su R Zhang J Chen J Ma X. Epitranscriptomics in Liver Disease: Basic Concepts and Therapeutic Potential. J Hepatol (2020) 73(3):664–79. doi: 10.1016/j.jhep.2020.04.009 32330603

[17] Li X Zhu P Ma S Song J Bai J Sun F. Chemical Pulldown Reveals Dynamic Pseudouridylation of the Mammalian Transcriptome. Nat Chem Biol (2015) 11(8):592–7. doi: 10.1038/nchembio.1836 26075521

[18] Schwartz S Bernstein DA Mumbach MR Jovanovic M Herbst RH Leon-Ricardo BX. Transcriptome-Wide Mapping Reveals Widespread Dynamic-Regulated Pseudouridylation of Ncrna and Mrna. Cell (2014) 159(1):148–62. doi: 10.1016/j.cell.2014.08.028 PMC4180118 25219674

[19] Jack K Bellodi C Landry DM Niederer RO Meskauskas A Musalgaonkar S. Rrna Pseudouridylation Defects Affect Ribosomal Ligand Binding and Translational Fidelity From Yeast to Human Cells. Mol Cell (2011) 44(4):660–6. doi: 10.1016/j.molcel.2011.09.017 PMC3222873 22099312

[20] Karijolich J Yi C Yu YT. Transcriptome-Wide Dynamics of RNA Pseudouridylation. Nat Rev Mol Cell Biol (2015) 16(10):581–5. doi: 10.1038/nrm4040 PMC5694666 26285676

[21] Li X Ma S Yi C. Pseudouridine: The Fifth RNA Nucleotide With Renewed Interests. Curr Opin Chem Biol (2016) 33:108–16. doi: 10.1016/j.cbpa.2016.06.014 27348156

[22] Dominissini D Nachtergaele S Moshitch-Moshkovitz S Peer E Kol N Ben-Haim MS. The Dynamic N(1)-Methyladenosine Methylome in Eukaryotic Messenger RNA. Nature (2016) 530(7591):441–6. doi: 10.1038/nature16998 PMC4842015 26863196

[23] Li X Xiong X Wang K Wang L Shu X Ma S. Transcriptome-Wide Mapping Reveals Reversible and Dynamic N(1)-Methyladenosine Methylome. Nat Chem Biol (2016) 12(5):311–6. doi: 10.1038/nchembio.2040 26863410

[24] Liu F Clark W Luo G Wang X Fu Y Wei J. ALKBH1-Mediated Trna Demethylation Regulates Translation. Cell (2016) 167(3):816–28.e16. doi: 10.1016/j.cell.2016.09.038 27745969 PMC5119773

[25] Wei J Liu F Lu Z Fei Q Ai Y He PC. Differential M(6)a, M(6)am, and M(1)a Demethylation Mediated by FTO in the Cell Nucleus and Cytoplasm. Mol Cell (2018) 71(6):973–85.e5. doi: 10.1016/j.molcel.2018.08.011 30197295 PMC6151148

[26] Dai X Wang T Gonzalez G Wang Y. Identification of YTH Domain-Containing Proteins as the Readers for N1-Methyladenosine in RNA. Anal Chem (2018) 90(11):6380–4. doi: 10.1021/acs.analchem.8b01703 PMC6157021 29791134

[27] Hauenschild R Tserovski L Schmid K Thuring K Winz ML Sharma S. The Reverse Transcription Signature of N-1-Methyladenosine in RNA-Seq Is Sequence Dependent. Nucleic Acids Res (2015) 43(20):9950–64. doi: 10.1093/nar/gkv895 PMC4787781 26365242

[28] Boulias K Toczydlowska-Socha D Hawley BR Liberman N Takashima K Zaccara S. Identification of the M(6)am Methyltransferase PCIF1 Reveals the Location and Functions of M(6)am in the Transcriptome. Mol Cell (2019) 75(3):631–43 e8. doi: 10.1016/j.molcel.2019.06.006 31279658 PMC6703822

[29] Akichika S Hirano S Shichino Y Suzuki T Nishimasu H Ishitani R. Cap-Specific Terminal N (6)-Methylation of RNA by an RNA Polymerase II-Associated Methyltransferase. Science (2019) 363(6423). doi: 10.1126/science.aav0080 30467178

[30] Sendinc E Valle-Garcia D Dhall A Chen H Henriques T Navarrete-Perea J. PCIF1 Catalyzes m6Am Mrna Methylation to Regulate Gene Expression. Mol Cell (2019) 75(3):620–30.e9. doi: 10.1016/j.molcel.2019.05.030 31279659 PMC6688901

[31] Elkon R Ugalde A Agami R. Alternative Cleavage and Polyadenylation: Extent, Regulation and Function. Nat Rev Genet (2013) 14(7):496–506. doi: 10.1038/nrg3482 23774734

[32] Tian B Manley JL. Alternative Polyadenylation of Mrna Precursors. Nat Rev Mol Cell Biol (2017) 18(1):18–30. doi: 10.1038/nrm.2016.116 27677860 PMC5483950

[33] Zhang LS Liu C Ma H Dai Q Sun HL Luo G. Transcriptome-Wide Mapping of Internal N(7)-Methylguanosine Methylome in Mammalian Mrna. Mol Cell (2019) 74(6):1304–16.e8. doi: 10.1016/j.molcel.2019.03.036 31031084 PMC6588483

[34] Lin S Liu Q Lelyveld VS Choe J Szostak JW Gregory RI. Mettl1/Wdr4-Mediated M(7)G Trna Methylome Is Required for Normal Mrna Translation and Embryonic Stem Cell Self-Renewal and Differentiation. Mol Cell (2018) 71(2):244–55 e5. doi: 10.1016/j.molcel.2018.06.001 29983320 PMC6086580

[35] Dai Q Moshitch-Moshkovitz S Han D Kol N Amariglio N Rechavi G. Nm-Seq Maps 2’-O-Methylation Sites in Human Mrna With Base Precision. Nat Methods (2017) 14(7):695–8. doi: 10.1038/nmeth.4294 PMC5712428 28504680

[36] Grozhik AV Jaffrey SR. Distinguishing RNA Modifications From Noise in Epitranscriptome Maps. Nat Chem Biol (2018) 14(3):215–25. doi: 10.1038/nchembio.2546 29443978

[37] Wu S Wang Y Wang J Li X Li J Ye K. Profiling of RNA Ribose Methylation in Arabidopsis Thaliana. Nucleic Acids Res (2021) 49(7):4104–19. doi: 10.1093/nar/gkab196 PMC8053127 33784398

[38] Squires JE Patel HR Nousch M Sibbritt T Humphreys DT Parker BJ. Widespread Occurrence of 5-Methylcytosine in Human Coding and Non-Coding RNA. Nucleic Acids Res (2012) 40(11):5023–33. doi: 10.1093/nar/gks144 PMC3367185 22344696

[39] Yang X Yang Y Sun BF Chen YS Xu JW Lai WY. 5-Methylcytosine Promotes Mrna Export - NSUN2 as the Methyltransferase and ALYREF as an M(5)C Reader. Cell Res (2017) 27(5):606–25. doi: 10.1038/cr.2017.55 PMC5594206 28418038

[40] Shen Q Zhang Q Shi Y Shi Q Jiang Y Gu Y. Tet2 Promotes Pathogen Infection-Induced Myelopoiesis Through Mrna Oxidation. Nature (2018) 554(7690):123–7. doi: 10.1038/nature25434 29364877

[41] Zou F Tu R Duan B Yang Z Ping Z Song X. Drosophila YBX1 Homolog YPS Promotes Ovarian Germ Line Stem Cell Development by Preferentially Recognizing 5-Methylcytosine Rnas. Proc Natl Acad Sci USA (2020) 117(7):3603–9. doi: 10.1073/pnas.1910862117 PMC7035628 32015133

[42] Li X Xiong X Yi C. Epitranscriptome Sequencing Technologies: Decoding RNA Modifications. Nat Methods (2016) 14(1):23–31. doi: 10.1038/nmeth.4110 28032622

[43] Arango D Sturgill D Alhusaini N Dillman AA Sweet TJ Hanson G. Acetylation of Cytidine in Mrna Promotes Translation Efficiency. Cell (2018) 175(7):1872–86 e24. doi: 10.1016/j.cell.2018.10.030 30449621 PMC6295233

[44] Sharma S Langhendries JL Watzinger P Kotter P Entian KD Lafontaine DL. Yeast Kre33 and Human NAT10 Are Conserved 18S Rrna Cytosine Acetyltransferases That Modify Trnas Assisted by the Adaptor Tan1/THUMPD1. Nucleic Acids Res (2015) 43(4):2242–58. doi: 10.1093/nar/gkv075 PMC4344512 25653167

[45] Licht K Jantsch MF. Rapid and Dynamic Transcriptome Regulation by RNA Editing and RNA Modifications. J Cell Biol (2016) 213(1):15–22. doi: 10.1083/jcb.201511041 27044895 PMC4828693

[46] Nishikura K. A-to-I Editing of Coding and Non-Coding Rnas by Adars. Nat Rev Mol Cell Biol (2016) 17(2):83–96. doi: 10.1038/nrm.2015.4 26648264 PMC4824625

[47] Blanc V Park E Schaefer S Miller M Lin Y Kennedy S. Genome-Wide Identification and Functional Analysis of Apobec-1-Mediated C-to-U RNA Editing in Mouse Small Intestine and Liver. Genome Biol (2014) 15(6):R79. doi: 10.1186/gb-2014-15-6-r79 24946870 PMC4197816

[48] Cao D Pizzorno G. Uridine Phosophorylase: An Important Enzyme in Pyrimidine Metabolism and Fluoropyrimidine Activation. Drugs Today (Barcelona Spain: 1998) (2004) 40(5):431–43. doi: 10.1358/dot.2004.40.5.850491 15319798

[49] Cao D Russell R Zhang D Leffert J Pizzorno G. Uridine Phosphorylase (-/-) Murine Embryonic Stem Cells Clarify the Key Role of This Enzyme in the Regulation of the Pyrimidine Salvage Pathway and in the Activation of Fluoropyrimidines. Cancer Res (2002) 62(8):2313–7. 11956089

[50] Brown C Alizadeh D Starr R Weng L Wagner J Naranjo A. Regression of Glioblastoma After Chimeric Antigen Receptor T-Cell Therapy. N Engl J Med (2016) 375(26):2561–9. doi: 10.1056/NEJMoa1610497 PMC5390684 28029927

[51] Scharping N Menk A Moreci R Whetstone R Dadey R Watkins S. The Tumor Microenvironment Represses T Cell Mitochondrial Biogenesis to Drive Intratumoral T Cell Metabolic Insufficiency and Dysfunction. Immunity (2016) 45(2):374–88. doi: 10.1016/j.immuni.2016.07.009 PMC5207350 27496732

[52] Xu S Tang L Li X Fan F Liu Z. Immunotherapy for Glioma: Current Management and Future Application. Cancer Lett (2020) 476:1–12. doi: 10.1016/j.canlet.2020.02.002 32044356

[53] Niu Y Lin Z Wan A Sun L Yan S Liang H. Loss-of-Function Genetic Screening Identifies ALDOA as an Essential Driver for Liver Cancer Cell Growth Under Hypoxia. Hepatol (Baltimore Md) (2021) 74(3):1461–79. doi: 10.1002/hep.31846 PMC8518375 33813748

[54] Yin H Zhang X Yang P Zhang X Peng Y Li D. RNA M6a Methylation Orchestrates Cancer Growth and Metastasis *via* Macrophage Reprogramming. Nat Commun (2021) 12(1):1394. doi: 10.1038/s41467-021-21514-8 33654093 PMC7925544

[55] Zhang B Wu Q Li B Wang D Wang L Zhou YL. M(6)a Regulator-Mediated Methylation Modification Patterns and Tumor Microenvironment Infiltration Characterization in Gastric Cancer. Mol Cancer (2020) 19(1):53. doi: 10.1186/s12943-020-01170-0 32164750 PMC7066851

[56] Du J Ji H Ma S Jin J Mi S Hou K. M6a Regulator-Mediated Methylation Modification Patterns and Characteristics of Immunity and Stemness in Low-Grade Glioma. Briefings Bioinf (2021) 22(5):bbab013. doi: 10.1093/bib/bbab013 33594424

[57] Yue X Lio CJ Samaniego-Castruita D Li X Rao A. Loss of TET2 and TET3 in Regulatory T Cells Unleashes Effector Function. Nat Commun (2019) 10(1):2011. doi: 10.1038/s41467-019-09541-y 31043609 PMC6494907

[58] Guo G Shi X Wang H Ye L Tong X Yan K. Epitranscriptomic N4-Acetylcytidine Profiling in CD4(+) T Cells of Systemic Lupus Erythematosus. Front Cell Dev Biol (2020) 8:842. doi: 10.3389/fcell.2020.00842 32984334 PMC7483482

[59] Nombela P Miguel-Lopez B Blanco S. The Role of M(6)a, M(5)C and Psi RNA Modifications in Cancer: Novel Therapeutic Opportunities. Mol Cancer (2021) 20(1):18. doi: 10.1186/s12943-020-01263-w 33461542 PMC7812662

[60] Bhat KPL Balasubramaniyan V Vaillant B Ezhilarasan R Hummelink K Hollingsworth F. Mesenchymal Differentiation Mediated by NF-Kappab Promotes Radiation Resistance in Glioblastoma. Cancer Cell (2013) 24(3):331–46. doi: 10.1016/j.ccr.2013.08.001 PMC3817560 23993863

[61] Li H Li J Chen L Qi S Yu S Weng Z. HERC3-Mediated SMAD7 Ubiquitination Degradation Promotes Autophagy-Induced EMT and Chemoresistance in Glioblastoma. Clin Cancer Res (2019) 25(12):3602–16. doi: 10.1158/1078-0432.CCR-18-3791 30862693

[62] Iwata R Hyoung Lee J Hayashi M Dianzani U Ofune K Maruyama M. ICOSLG-Mediated Regulatory T-Cell Expansion and IL-10 Production Promote Progression of Glioblastoma. Neuro-Oncology (2020) 22(3):333–44. doi: 10.1093/neuonc/noz204 PMC7442411 31634400

[63] Xu J Zhang Z Qian M Wang S Qiu W Chen Z. Cullin-7 (CUL7) Is Overexpressed in Glioma Cells and Promotes Tumorigenesis *via* NF-κb Activation. J Exp Clin Cancer Res (2020) 39(1):59. doi: 10.1186/s13046-020-01553-7 32252802 PMC7132976

[64] Zhang Z Xu J Chen Z Wang H Xue H Yang C. Transfer of Microrna *via* Macrophage-Derived Extracellular Vesicles Promotes Proneural-to-Mesenchymal Transition in Glioma Stem Cells. Cancer Immunol Res (2020) 8(7):966–81. doi: 10.1158/2326-6066.CIR-19-0759 32350000

[65] Liu X Fu Y Huang J Wu M Zhang Z Xu R. ADAR1 Promotes the Epithelial-to-Mesenchymal Transition and Stem-Like Cell Phenotype of Oral Cancer by Facilitating Oncogenic Microrna Maturation. J Exp Clin Cancer Res: CR (2019) 38(1):315. doi: 10.1186/s13046-019-1300-2 31315644 PMC6637647

[66] Liu X Chen D Chen H Wang W Liu Y Wang Y. YB1 Regulates Mir-205/200b-ZEB1 Axis by Inhibiting Microrna Maturation in Hepatocellular Carcinoma. Cancer Commun (2021) 41(7):576–95. doi: 10.1002/cac2.12164 PMC8286141 34110104

[67] Bindea G Mlecnik B Tosolini M Kirilovsky A Waldner M Obenauf A. Spatiotemporal Dynamics of Intratumoral Immune Cells Reveal the Immune Landscape in Human Cancer. Immunity (2013) 39(4):782–95. doi: 10.1016/j.immuni.2013.10.003 24138885

[68] Yoshihara K Shahmoradgoli M Martínez E Vegesna R Kim H Torres-Garcia W. Inferring Tumour Purity and Stromal and Immune Cell Admixture From Expression Data. Nat Commun (2013) 4:2612. doi: 10.1038/ncomms3612 24113773 PMC3826632

[69] Verhaak R Hoadley K Purdom E Wang V Qi Y Wilkerson M. Integrated Genomic Analysis Identifies Clinically Relevant Subtypes of Glioblastoma Characterized by Abnormalities in PDGFRA, IDH1, EGFR, and NF1. Cancer Cell (2010) 17(1):98–110. doi: 10.1016/j.ccr.2009.12.020 20129251 PMC2818769

[70] Jiang T Nam DH Ram Z Poon WS Wang J Boldbaatar D. Clinical Practice Guidelines for the Management of Adult Diffuse Gliomas. Cancer Lett (2021) 499:60–72. doi: 10.1016/j.canlet.2020.10.050 33166616

[71] Chen DS Mellman I. Elements of Cancer Immunity and the Cancer-Immune Set Point. Nature (2017) 541(7637):321–30. doi: 10.1038/nature21349 28102259

[72] Qian M Wang S Guo X Wang J Zhang Z Qiu W. Hypoxic Glioma-Derived Exosomes Deliver Microrna-1246 to Induce M2 Macrophage Polarization by Targeting TERF2IP *via* the STAT3 and NF-κb Pathways. Oncogene (2020) 39(2):428–42. doi: 10.1038/s41388-019-0996-y 31485019

[73] Xu J Zhang J Zhang Z Gao Z Qi Y Qiu W. Hypoxic Glioma-Derived Exosomes Promote M2-Like Macrophage Polarization by Enhancing Autophagy Induction. Cell Death Dis (2021) 12(4):373. doi: 10.1038/s41419-021-03664-1 33828078 PMC8026615

[74] Hugo W Zaretsky JM Sun L Song C Moreno BH Hu-Lieskovan S. Genomic and Transcriptomic Features of Response to Anti-PD-1 Therapy in Metastatic Melanoma. Cell (2016) 165(1):35–44. doi: 10.1016/j.cell.2016.02.065 26997480 PMC4808437

[75] Ayers M Lunceford J Nebozhyn M Murphy E Loboda A Kaufman DR. IFN-Gamma-Related Mrna Profile Predicts Clinical Response to PD-1 Blockade. J Clin Invest (2017) 127(8):2930–40. doi: 10.1172/JCI91190 PMC5531419 28650338

[76] Phillips HS Kharbanda S Chen R Forrest WF Soriano RH Wu TD. Molecular Subclasses of High-Grade Glioma Predict Prognosis, Delineate a Pattern of Disease Progression, and Resemble Stages in Neurogenesis. Cancer Cell (2006) 9(3):157–73. doi: 10.1016/j.ccr.2006.02.019 16530701

[77] Behnan J Finocchiaro G Hanna G. The Landscape of the Mesenchymal Signature in Brain Tumours. Brain (2019) 142(4):847–66. doi: 10.1093/brain/awz044 PMC6485274 30946477

[78] McGranahan N Furness AJS Rosenthal R Ramskov S Lyngaa R Saini SK. Clonal Neoantigens Elicit T Cell Immunoreactivity and Sensitivity to Immune Checkpoint Blockade. Science (2016) 351(6280):1463. doi: 10.1126/science.aaf1490 26940869 PMC4984254

[79] Cristescu R Mogg R Ayers M Albright A Murphy E Yearley J. Pan-Tumor Genomic Biomarkers for PD-1 Checkpoint Blockade-Based Immunotherapy. Science (2018) 362(6411):eaar3593. doi: 10.1126/science.aar3593 PMC6718162 30309915

[80] Goodman AM Kato S Bazhenova L Patel SP Frampton GM Miller V. Tumor Mutational Burden as an Independent Predictor of Response to Immunotherapy in Diverse Cancers. Mol Cancer Ther (2017) 16(11):2598–608. doi: 10.1158/1535-7163.MCT-17-0386 PMC5670009 28835386

[81] Mariathasan S Turley SJ Nickles D Castiglioni A Yuen K Wang Y. Tgfbeta Attenuates Tumour Response to PD-L1 Blockade by Contributing to Exclusion of T Cells. Nature (2018) 554(7693):544–8. doi: 10.1038/nature25501 PMC6028240 29443960

[82] Balar AV Galsky MD Rosenberg JE Powles T Petrylak DP Bellmunt J. Atezolizumab as First-Line Treatment in Cisplatin-Ineligible Patients With Locally Advanced and Metastatic Urothelial Carcinoma: A Single-Arm, Multicentre, Phase 2 Trial. Lancet (2017) 389(10064):67–76. doi: 10.1016/S0140-6736(16)32455-2 27939400 PMC5568632

[83] Yang W Soares J Greninger P Edelman EJ Lightfoot H Forbes S. Genomics of Drug Sensitivity in Cancer (GDSC): A Resource for Therapeutic Biomarker Discovery in Cancer Cells. Nucleic Acids Res (2013) 41(Database issue):D955–61. doi: 10.1093/nar/gks1111 PMC3531057 23180760

[84] Joyce J Fearon D. T Cell Exclusion, Immune Privilege, and the Tumor Microenvironment. Science (New York NY) (2015) 348(6230):74–80. doi: 10.1126/science.aaa6204 25838376

[85] Wang Z Zhang H Xu S Liu Z Cheng Q. The Adaptive Transition of Glioblastoma Stem Cells and Its Implications on Treatments. Signal Transduct Target Ther (2021) 6(1):124. doi: 10.1038/s41392-021-00491-w 33753720 PMC7985200

