# Supplementary material for: The Non-N6-Methyladenosine Epitranscriptome Patterns and Characteristics of Tumor Microenvironment Infiltration and Mesenchymal Transition in Glioblastoma
Source: Front Immunol. 2022 Jan 26;12:809808. doi: 10.3389/fimmu.2021.809808 (PMC8825368; doi:10.3389/fimmu.2021.809808)
Supplement: Supplementary file 1 [file DataSheet_1.pdf]

Figure S1. Overview of study design and prognostic analysis of 32 non-m⁶A RNA modification regulators.

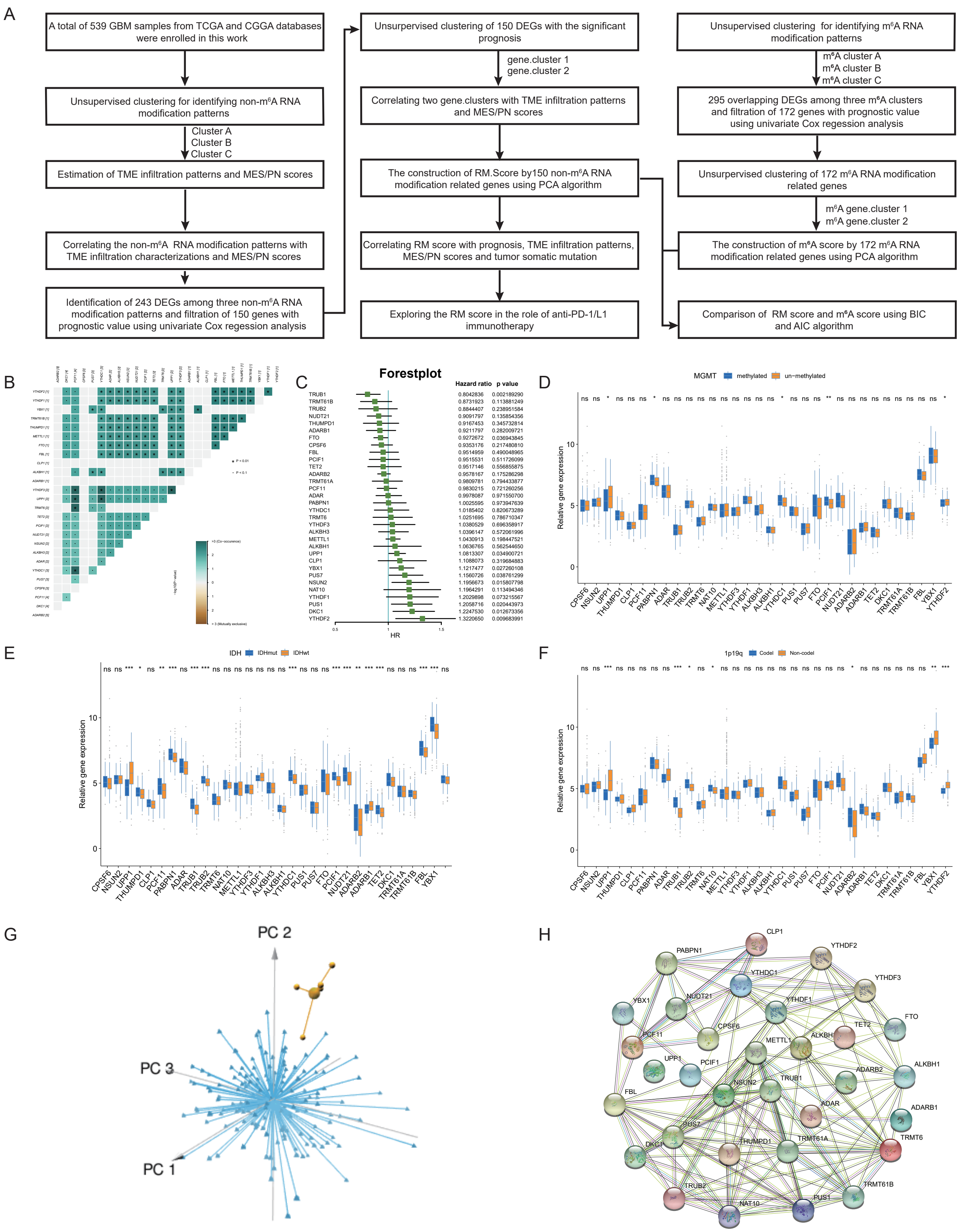

(A) Flow chart of this study. (B) Mutation co-occurrence and exclusion analyses for 32 non-m⁶A RNA modification regulators. Co-occurrence, green; Exclusion, purple. (C) The prognostic analyses for 32 non-m⁶A RNA modification regulators using a univariate Cox regression model. A hazard ratio >1 indicated risk factors for survival, and a hazard ratio <1 indicated protective factors for survival. (D) The expression of 32 non-m⁶A RNA modification regulators between MGMT promoter methylation and unmethylation. (E) Expression of 32 non-m⁶A RNA modification genes between the IDH mutant and IDH wild-type groups. (F) Differences in the expression of 32 non-m⁶A RNA modification genes between 1p19q codeletion and non-codeletion. (G) Principal component analysis of the expression profiles of 32 non-m⁶A RNA modification regulators to distinguish tumors from normal samples in the TCGA-GBM cohort. Two subgroups without intersection were identified, meaning that the GBM and normal samples were well distinguished based on the expression profiles of the 32 non-m⁶A RNA modification regulators. (H) The protein-protein interaction among 32 non-m⁶A RNA modification regulators.

Figure S2. Unsupervised clustering of 32 non-m⁶A RNA modification regulators.

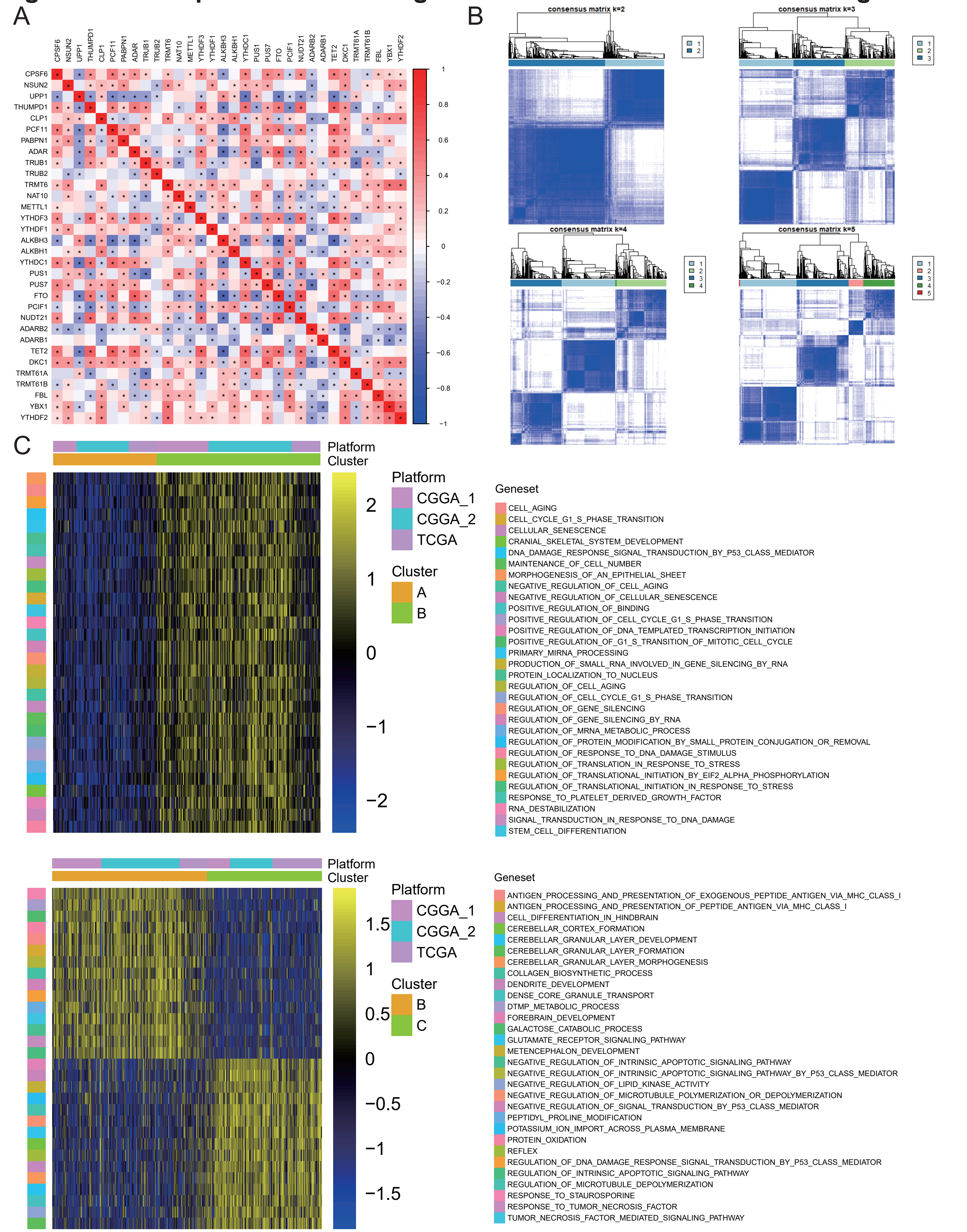

(A) The correlation between each non-m⁶A RNA modification regulator using Spearman analyses. A negative correlation is marked with red, and a positive correlation is marked with blue. (B) Consensus matrices of the 539 GBM samples for k = 2-5. (C) GSVA enrichment analysis showing the activation states of GO biological pathways in three distinct non-m⁶A RNA modification patterns. A heatmap was used to visualize these biological processes; yellow represents activated pathways, and blue represents inhibited pathways. Upper Cluster_A vs. Cluster_B; lower Cluster_B vs. Cluster_C.

Figure S3. Correlation between non-m⁶A RNA modification regulators and TME infiltration cells, MES scores, and PN scores.

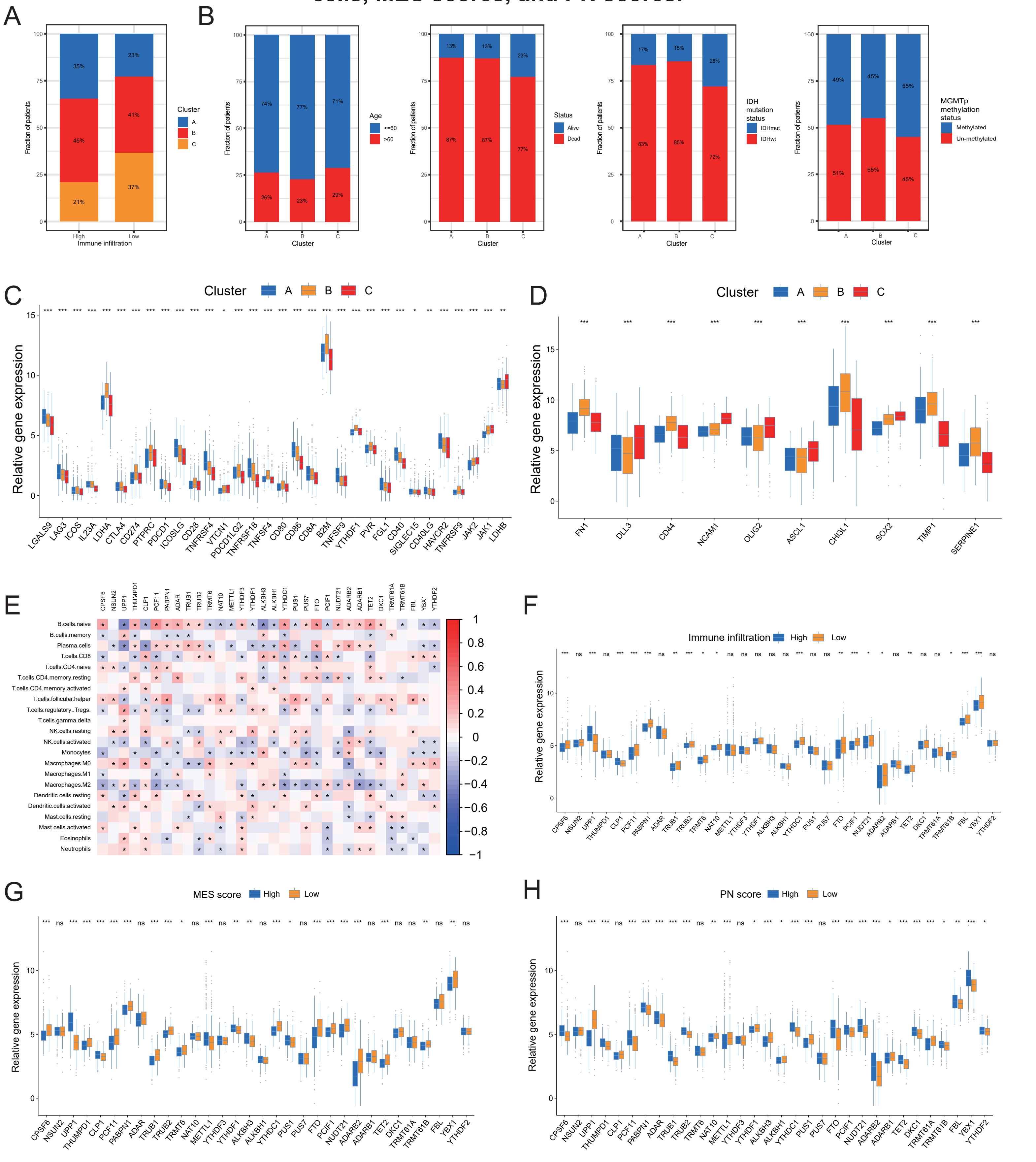

(A) The proportion of three non-m⁶A RNA modification patterns in the low and high immune infiltration groups. (B) The proportion of groups divided by age, survival status, IDH mutant status and MGMT promoter methylation status in three non-m⁶A RNA modification clusters. (C) The expression of immune checkpoint-related genes in three non-m⁶A RNA modification patterns. (D) The expression of MES/PN markers in three non-m⁶A RNA modification patterns. (E) The correlation between each non-m⁶A RNA modification regulator and each TME infiltration cell type using Spearman analyses. A negative correlation is marked with blue, and a positive correlation is marked with red. (F) The expression of 32 non-m⁶A RNA modification regulators in the low and high immune infiltration groups. (G) The expression of 32 non-m⁶A RNA modification regulators in the low and high MES score groups. (H) The expression of 32 non-m⁶A RNA modification regulators in the low and high PN score groups.

Figure S4. The roles of UPP1 in TME infiltration and MES transition.

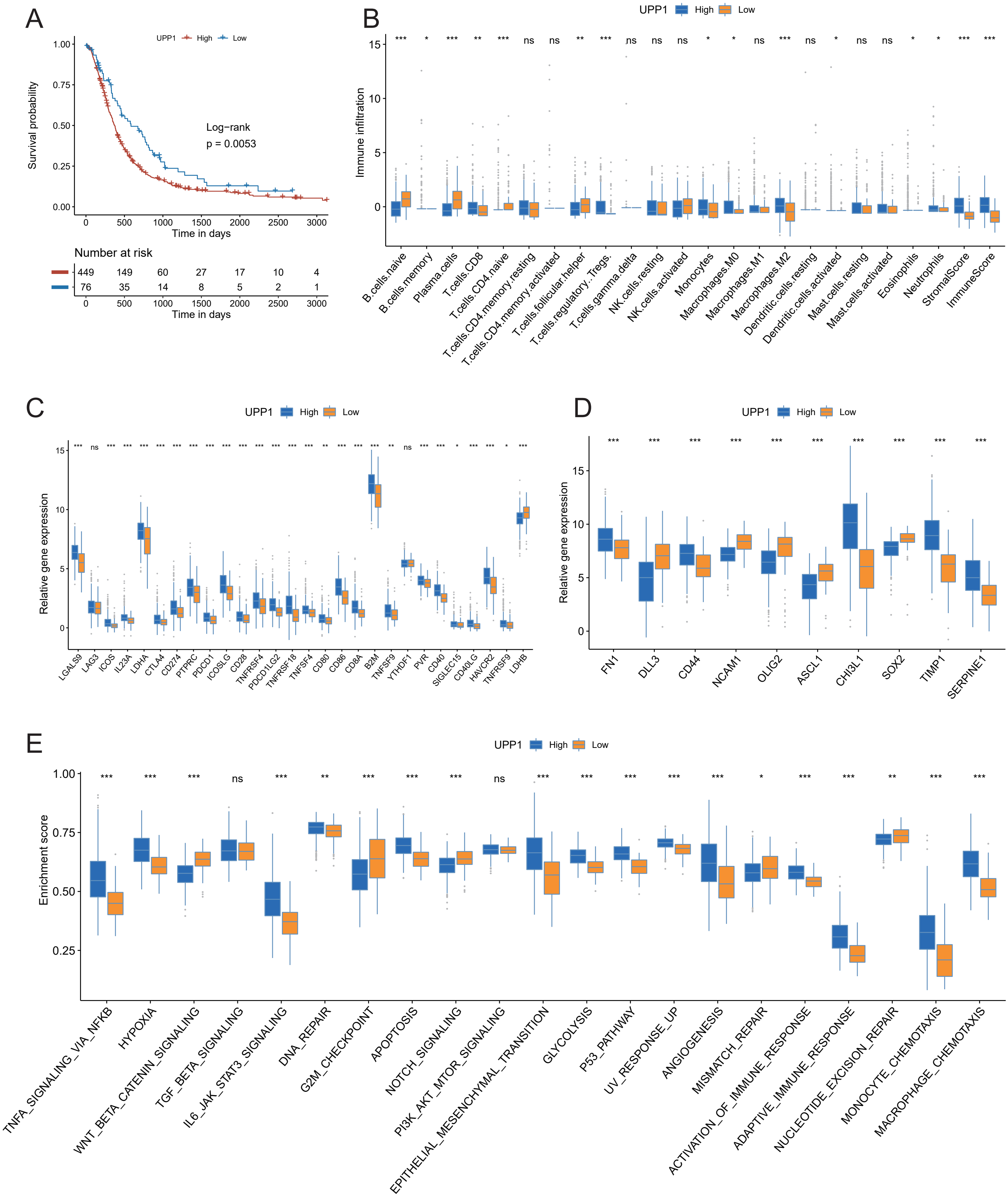

(A) Survival analyses for patients with low or high UPP1 expression using Kaplan-Meier curves (P < 0.0001, log-rank test). (B) The abundance of each TME-infiltrating cell, stromal scores and immune scores for patients with low or high UPP1 expression. (C) The expression of immune checkpoint-related genes in patients with low or high UPP1 expression. (D) The expression of MES/PN markers in the low or high UPP1 expression groups. (E) Differences in the TME infiltration and MES transition pathways among the low and high UPP1 expression groups. The asterisks represent the statistical p value (* P < 0.05; ** P < 0.01; *** P < 0.001).

Figure S5. The roles of FTO in TME infiltration and MES transition.

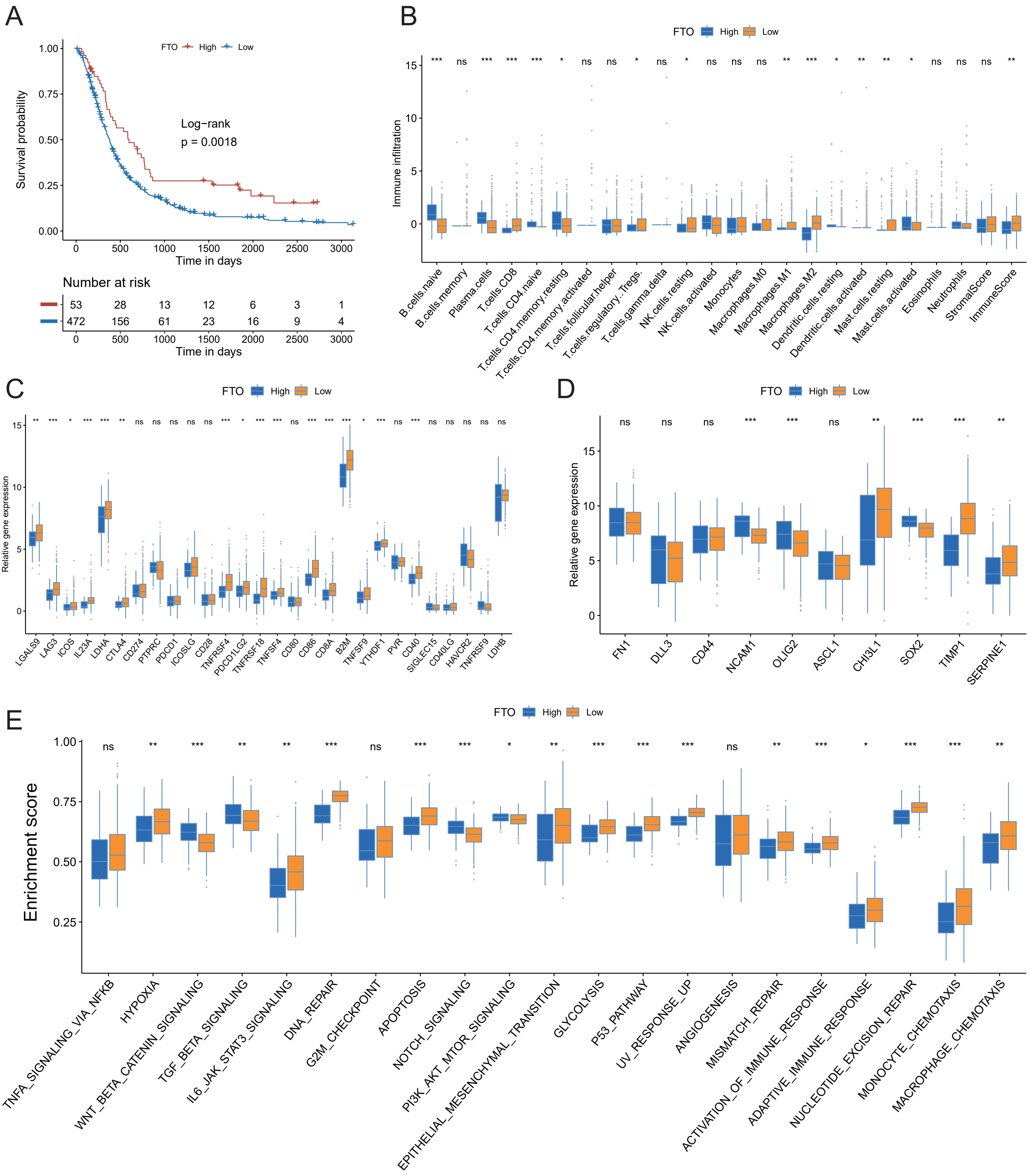

(A) Survival analyses for patients with low or high FTO expression using Kaplan-Meier curves (P < 0.0001, log-rank test). (B) The abundance of each TME-infiltrating cell, stromal scores and immune scores for patients with low or high FTO expression. (C) The expression of immune checkpoint-related genes in patients with low or high FTO expression. (D) The expression of MES/PN markers in the low or high FTO expression groups. (E) Differences in the TME infiltration and MES transition pathways among the low or high FTO expression groups. The asterisks represent the statistical p value (* P < 0.05; ** P < 0.01; *** P < 0.001).

Figure S6. Construction of differentially expressed non-m⁶A RNA modification gene signatures.

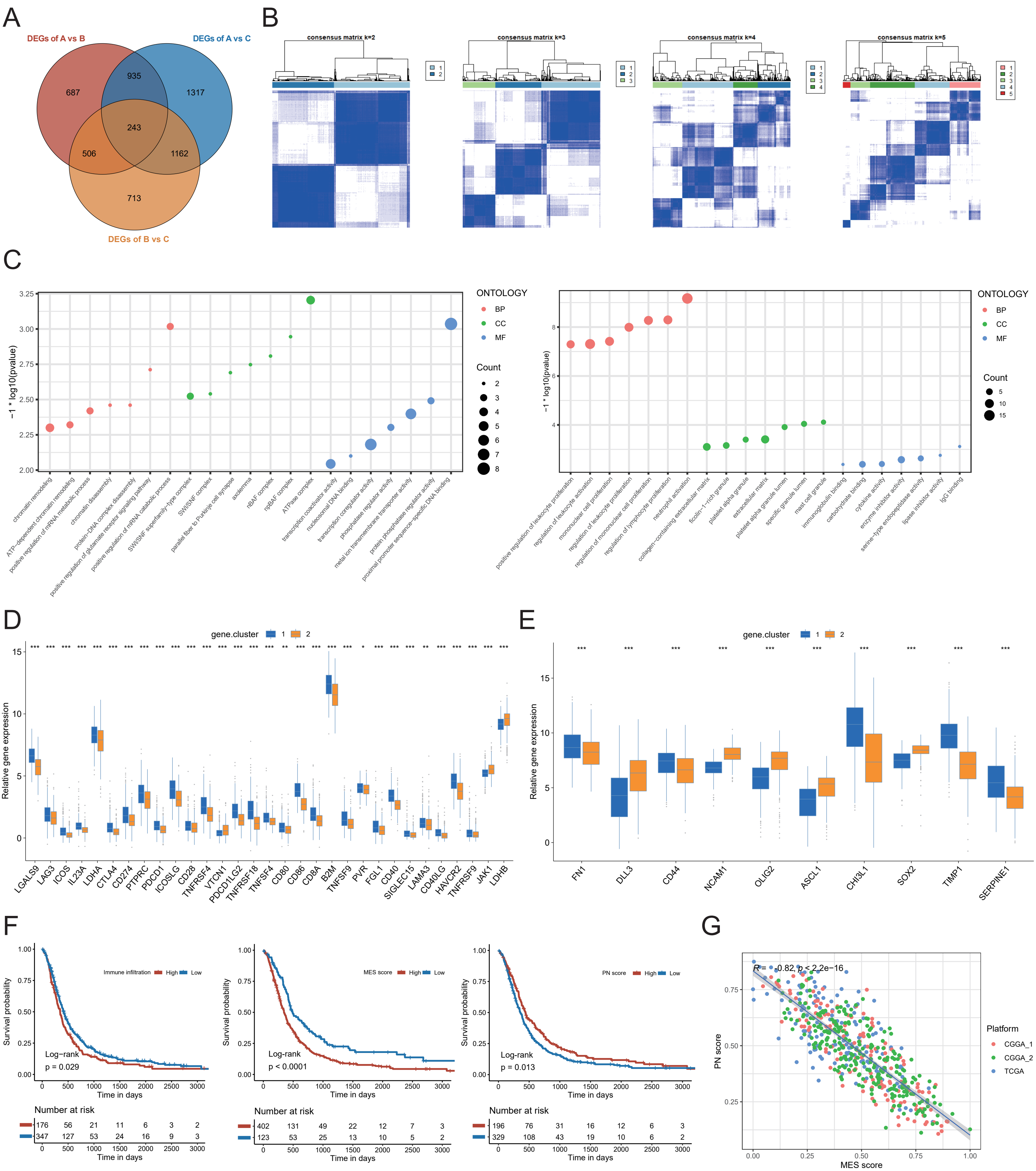

(A) A total of 243 overlapping DEGs are shown in the Venn diagram. (B) Consensus matrices of the 539 GBMs for k = 2-5. (C) GO enrichment analysis of the two groups of DEGs: left, genes upregulated in gene.cluster_2; right, genes upregulated in gene.cluster_1. The x axis indicates the number of genes within each GO term. (D) The expression of immune checkpoint-related genes in two gene.clusters. (E) The expression of MES/PN markers in two gene.clusters. (F) Left, survival analyses for low or high immune infiltration groups using Kaplan-Meier curves ($P < 0.029$, log-rank test); Middle, survival analyses for patients with low or high MES scores using Kaplan-Meier curves ($P < 0.0001$, log-rank test); Right, survival analyses for patients with low or high PN scores using Kaplan-Meier curves ($P < 0.013$, log-rank test). (G) Scatterplots depicting the negative correlation between MES score and PN score ($r = -0.82$, $P < 2.2e-16$, Spearman correlation analysis).

Figure S7. Predictive function of the RM score on GBM characteristics.

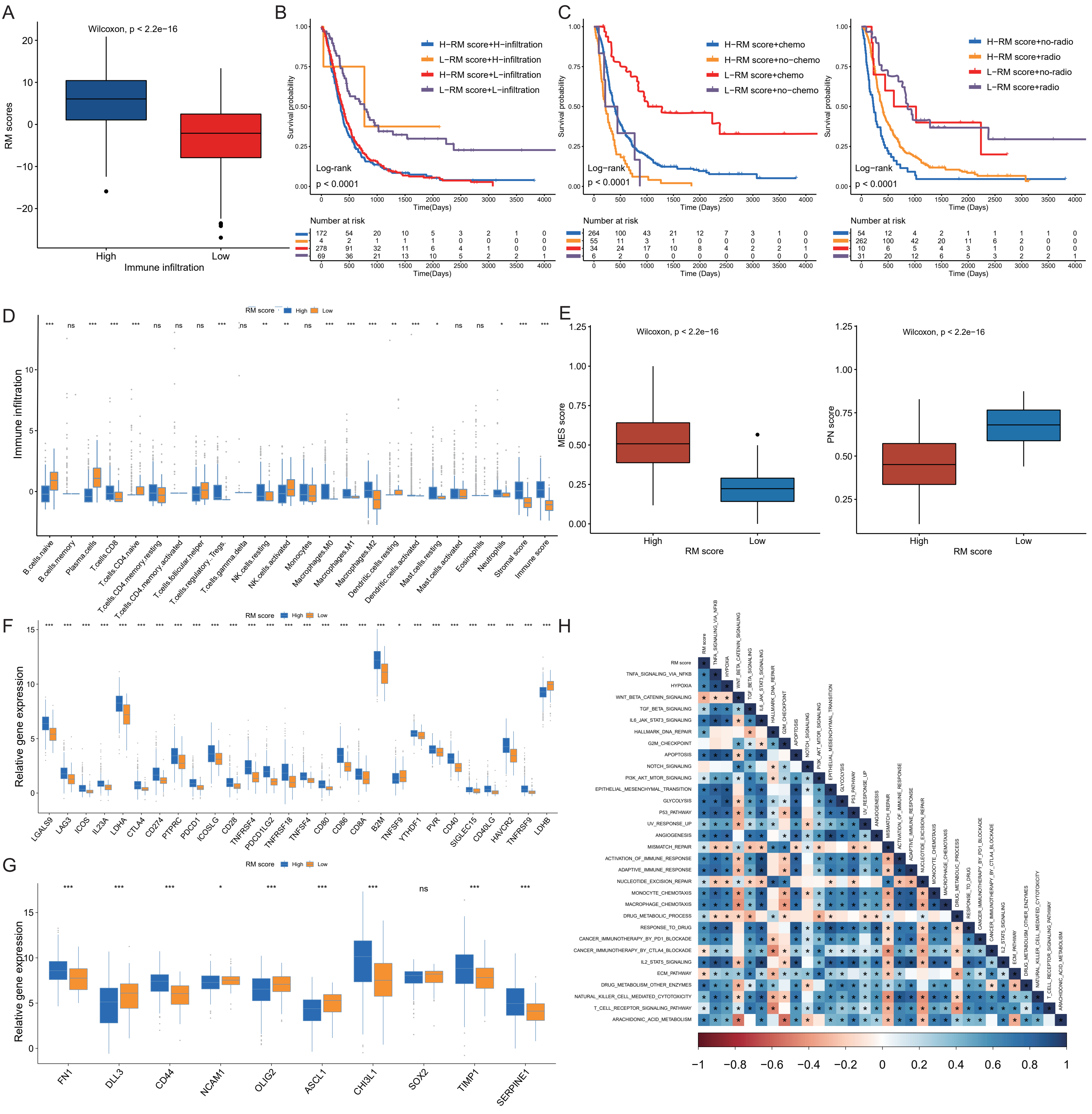

(A) Differences in RM scores among the two immune infiltration groups (P < 2.2e-16, Kruskal-Wallis test). (B) Survival analyses of patient subgroups stratified by RM score as well as immune infiltration status. (C) Survival analyses of GBM subgroups stratified by RM score and chemotherapy or radio-therapy. (D) The quantized values of TME-infiltrating cells, stromal scores and immune scores between GBMs associated with high and low RM scores. (E) Differences in MES/PN scores among the high RM score and low RM score groups. (F) The expression of immune checkpoint-related genes in the high RM score and low RM score groups. (G) The expression of MES/PN markers in the high RM score and low RM score groups. (H) Correlations between RM score and the TME infiltration and MES transition pathways using Spearman analysis.

Figure S8. Prognostic value of the RM score in GBM cohorts and the relationship between RM score and response to immunotherapy.

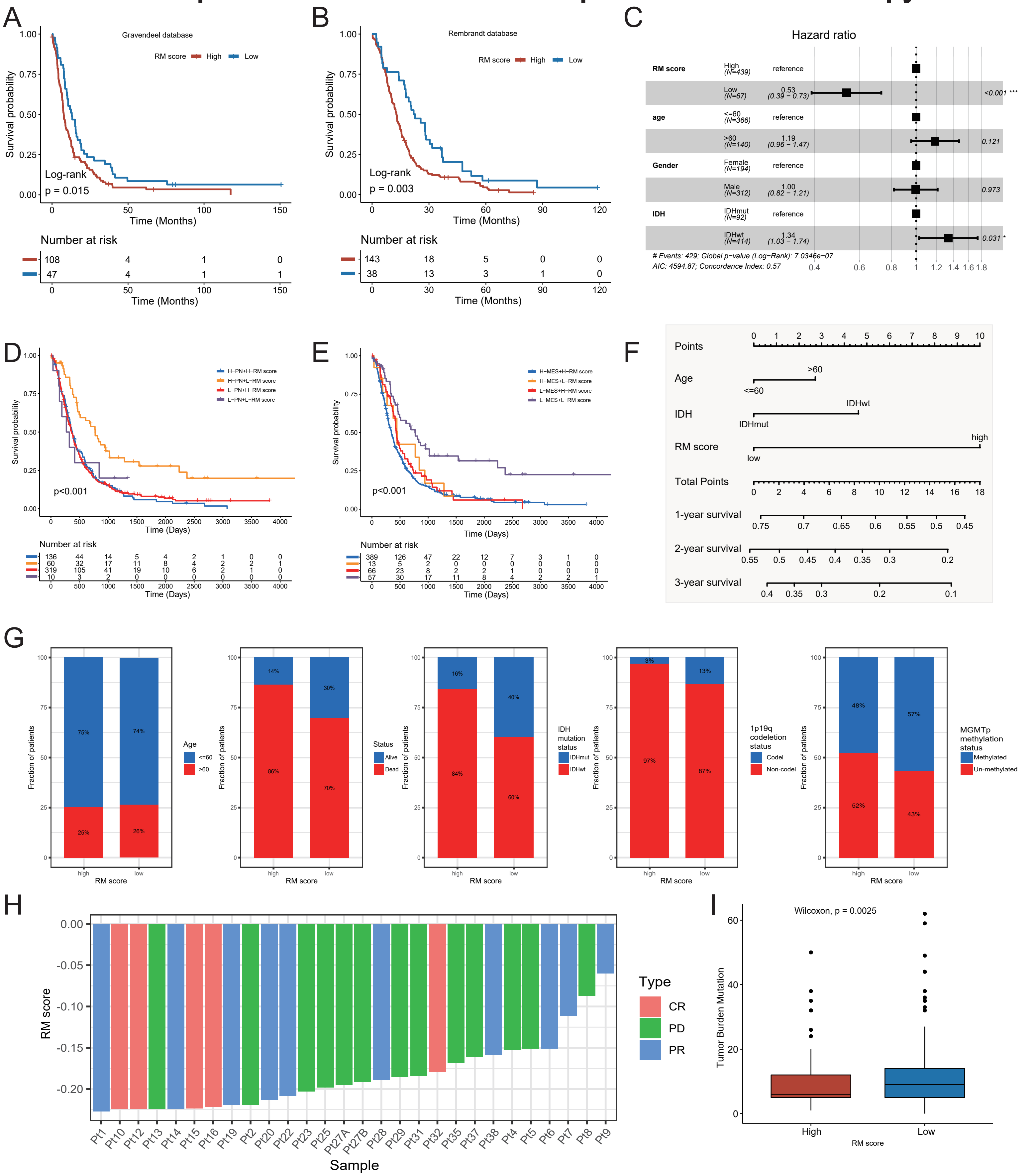

(A) Survival analyses for low or high RM score groups in the Gravendeel GBM cohort using Kaplan-Meier curves (P < 0.015, log-rank test). (B) Survival analyses for the low or high RM score groups in the Rembrandt GBM cohort using Kaplan-Meier curves (P < 0.003, log-rank test). (C) Multivariate Cox regression model analysis, which included the factors of RM score, patient age, sex and IDH status. (D) Survival analyses for GBM patients stratified by both RM and PN scores using Kaplan-Meier curves. H, high; L, Low; (P < 0.001, log-rank test). (E) Survival analyses for GBM patients stratified by both RM score and MES score using Kaplan-Meier curves. H, high; L, Low; (P < 0.001, Log-rank test). (F) The nomogram to predict 1-, 2- and 3-year survival was created based on RM score, patient age, and IDH status. (G) The proportion of groups divided by age, survival status, IDH mutant status, 1p19q codeletion status and MGMT promoter methylation status in the low and high RM score groups. (H) The correlation of RM score with clinical response to anti-PD-1 immunotherapy. Pt, patients. PD, green; PR, blue; CR, red. (I) TMB difference between the high and low RM score groups (P = 0.0025, Wilcoxon test).

Figure S9. The correlation between RM scores and TME levels in patients with PN and MES subtypes

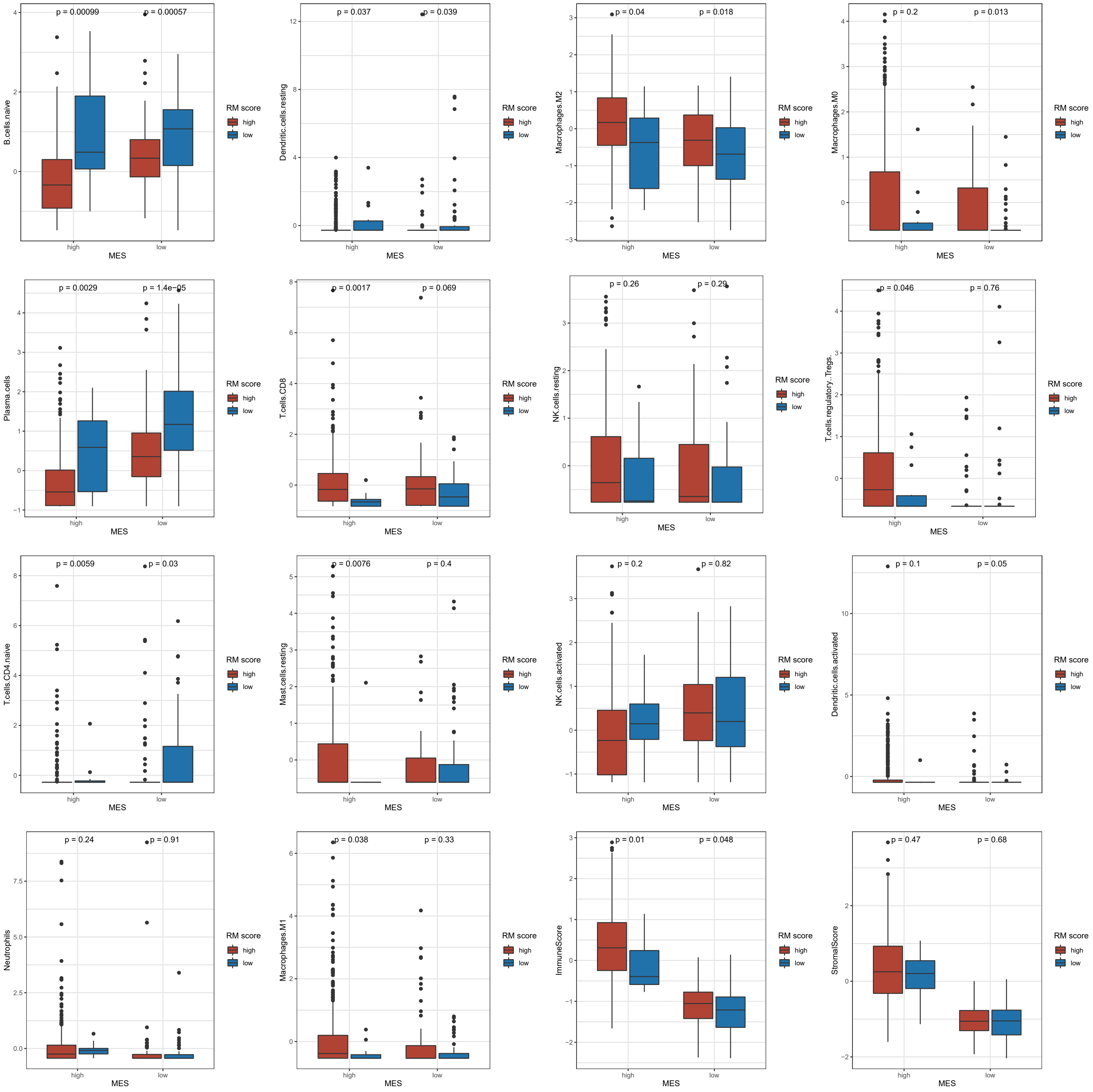

Figure S10. Construction of the m^{6}A score and comparison of the m^{6}A and RM scores.

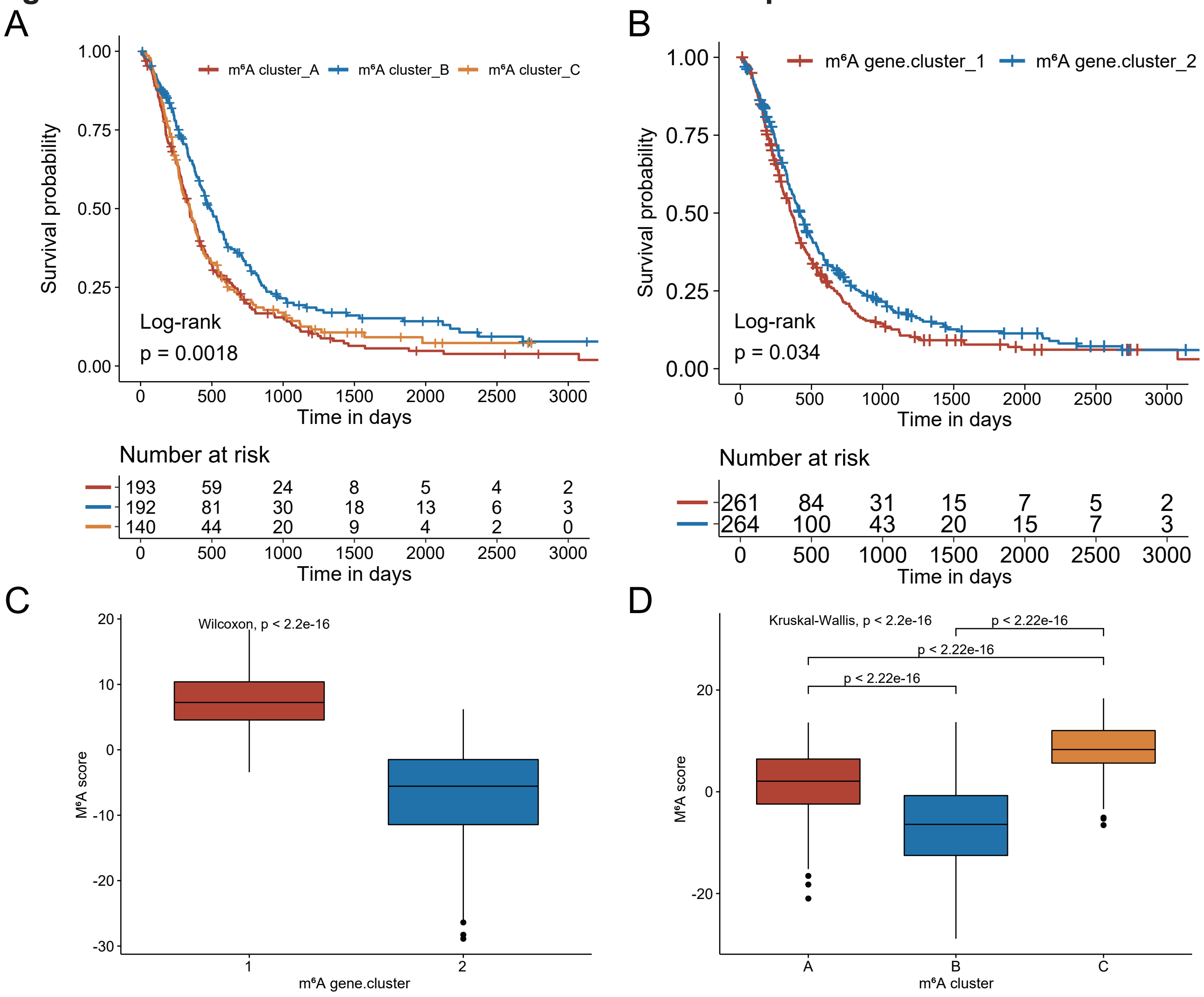

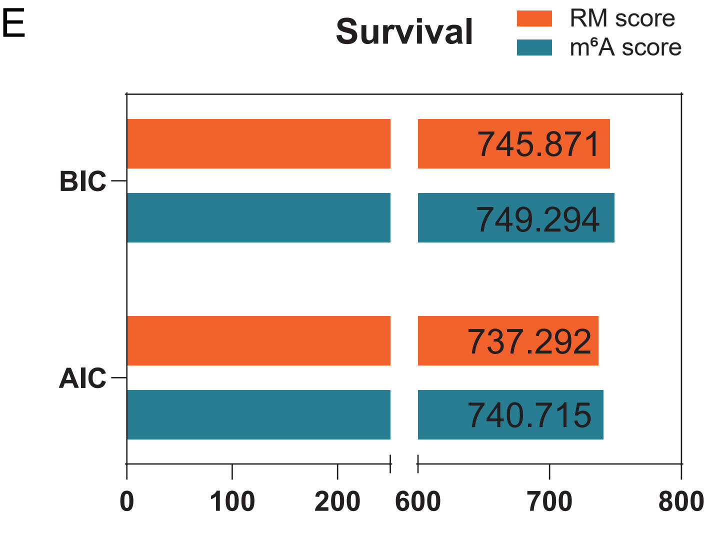

(A)Survival analyses for the three m^{6}A RNA modification patterns based on 539 GBM patients. Kaplan-Meier curves with a log-rank p value of 0.0018 showed a significant survival difference among the three non-m^{6}A RNA modification patterns. Cluster_B showed a significantly better survival prognosis than the other two clusters. (B) Survival analyses for the m^{6}A gene.cluster_1 and m^{6}A gene.cluster_2 patient groups using Kaplan-Meier curves (P = 0.034, log-rank test). (C, D) Differences in m^{6}A scores between two m^{6}A gene.clusters and three m^{6}A patterns. (E) Comparison of the value of the RM and m^{6}A scores in predicting patient outcomes.

Figure S11. RM score correlates with the immune infiltration and survival prognosis in Pan-cancer.

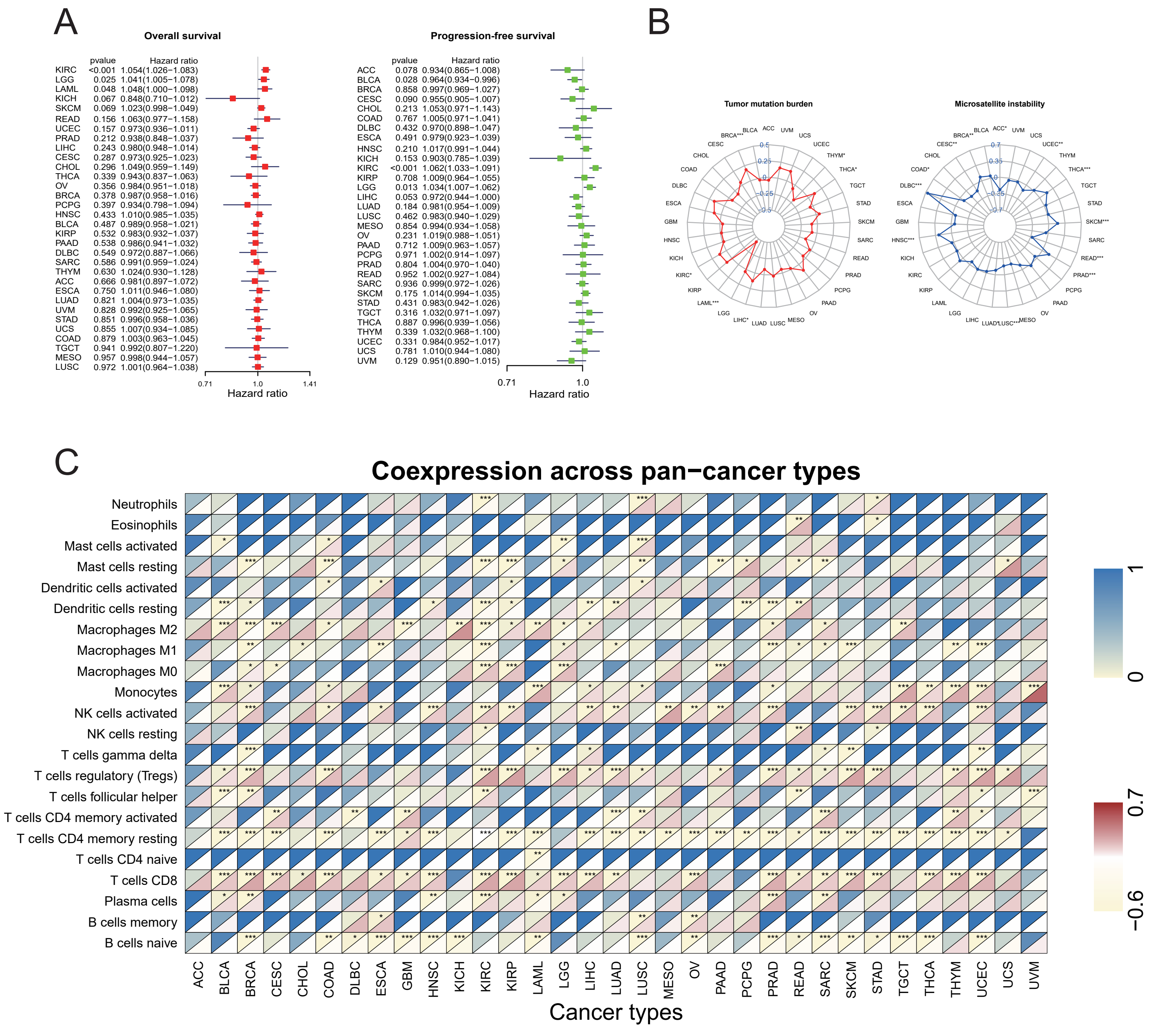

(A) The forest plots of univariate Cox regression analyses. (B) Radar chart of the correlation between RM score and TMB and MSI. (C) The immune cell infiltration was calculated by CIBERSORT algorithm. The upper part of each grid showed the P-value, and the bottom part showed the correlation coefficient.
